# Human gut microbiome: Therapeutic opportunities for metabolic syndrome—Hype or hope?

**DOI:** 10.1002/edm2.436

**Published:** 2023-09-28

**Authors:** Angela Horvath, Kristina Zukauskaite, Olha Hazia, Irina Balazs, Vanessa Stadlbauer

**Affiliations:** ^1^ Medical University of Graz Graz Austria; ^2^ Center for Biomarker Research in Medicine (CBmed) Graz Austria; ^3^ Life Sciences Centre Vilnius University Vilnius Lithuania

**Keywords:** diet, faecal microbiota transplantation, metabolic syndrome, microbiome, obesity, prebiotic, probiotic

## Abstract

Shifts in gut microbiome composition and metabolic disorders are associated with one another. Clinical studies and experimental data suggest a causal relationship, making the gut microbiome an attractive therapeutic goal. Diet, intake of probiotics or prebiotics and faecal microbiome transplantation (FMT) are methods to alter a person's microbiome composition. Although FMT may allow establishing a proof of concept to use microbiome modulation to treat metabolic disorders, studies show mixed results regarding the effects on metabolic parameters as well as on the composition of the microbiome. This review summarizes the current knowledge on diet, probiotics, prebiotics and FMT to treat metabolic diseases, focusing on studies that also report alterations in microbiome composition. Furthermore, clinical trial results on the effects of common drugs used to treat metabolic diseases are synopsized to highlight the bidirectional relationship between the microbiome and metabolic diseases. In conclusion, there is clear evidence that microbiome modulation has the potential to influence metabolic diseases; however, it is not possible to distinguish which intervention is the most successful. In addition, a clear commitment from all stakeholders is necessary to move forward in the direction of developing targeted interventions for microbiome modulation.

## Graphical abstract

We reviewed clinical trials using diet, prebiotics, probiotics and faecal microbiota transplantation to treat metabolic syndrome via microbiome modulation. We also reviewed the effect of the most important drugs to treat metabolic syndrome on the microbiome. We conclude that microbiome modulation is a valuable therapeutic tool in metabolic syndrome but more research is needed to define the most useful therapeutic intervention.

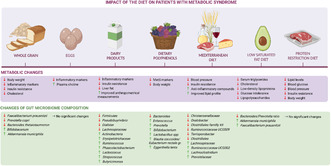

## 1 INTRODUCTION

Obesity and the metabolic syndrome (MetS) are on the rise in many parts of the world. They lead to a considerable burden of disease due to complications, such as cardiovascular disorders, cancer, dementia or fertility disorders as well as being associated with socio‐economic disadvantages.^1^ Associations between the gut microbiome composition and metabolic diseases have been known for almost 20 years.^2^ Obesity and its relation to shifts in the microbiome composition were among the first described associations when culture‐independent techniques to study the complex ecosystem of the gut microbiome were developed.^3^ Starting from a description of shifts in microbiome composition on the phylum level, a large body of literature has evolved that describes the bidirectional interaction between the gut microbiome and human metabolism.^4^

In human subject research, an association between the gut microbiome composition and obesity has widely been demonstrated. A causal relationship was first suggested by showing that a relative decrease in Bacteroides is associated with obesity, whereas a diet leading to weight loss leads to an increase in Bacteroides.^5^ Data from in vitro systems and animal models suggest that diet or drugs (e.g. antibiotics) alter the microbiome and that this dysbiosis is mechanistically involved in disrupting molecular metabolism as well as signalling through bacterial metabolites (e.g. bile acids). This impacts energy intake and leads to metabolic disorders. For example, diets high in fat and glucose lead to increased gut permeability, translocation of bacterial products, low‐grade inflammatory response and insulin resistance.^6^, ^7^ Bile acids are bidirectionally interacting with the microbiome—the microbiome influences bile acid composition and bile acids shape the microbiome. In obesity, altering bile acid composition via a reduction of microbial diversity through an antibiotic leads to increased insulin resistance.^8^ Altered microbiome composition, leading to altered bile acid metabolism and signalling might also contribute to fibrogenesis, liver injury and tumorigenesis in non‐alcoholic fatty liver disease.^9^ Host genetics also play a role since immune control of the microbiome is beneficial to microbial populations that constrain lipid metabolism to prevent metabolic syndrome.^10^ However, these findings cannot be readily translated to treat or prevent human obesity yet, in part due to limitations in the experimental systems, thereby preventing the establishment of clear causality. Much of the current findings in the research of obesity and microbiome remain at the level of associations.

**FIGURE 1:**
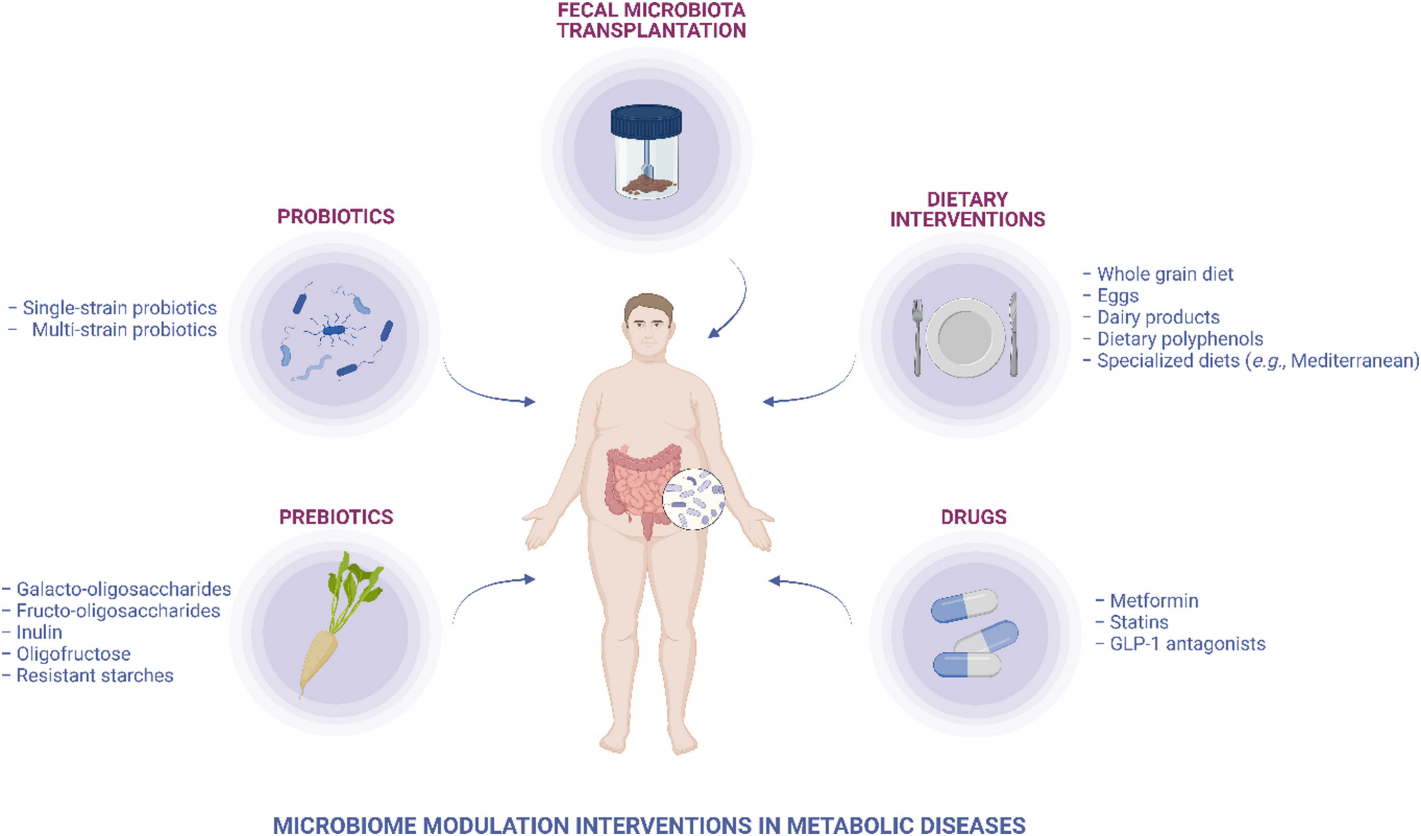
Our review summarizes the effect of diet, prebiotics, probiotics, fecal microbiome transplantation and drugs on the gut microbiome in metabolic diseases.

In this scoping review, we aimed to summarize the clinical evidence on the potential of diet, prebiotics, probiotics, faecal microbiome transplantation and selected drugs to treat and/or prevent metabolic disorders via microbiome modulation (Figure 1).

## 2 METHODS

A literature search was performed by all authors primarily in Pubmed between August and December 2022 using the key words ‘gut microbiome’, ‘microbiota’, ‘obesity’, ‘overweight’, ‘metabolic syndrome’, ‘diabetes’, ‘probiotic’, ‘prebiotic’, ‘synbiotic’, ‘galacto‐oligosaccharides’, ‘fructo‐oligosaccharides’, ‘inulin’, ‘postbiotic’, ‘fecal microbiota transplantation’, ‘diet’, ‘metformin’, ‘statin’ and ‘GLP‐1 agonist’ in various combinations. Reference lists from the retrieved publications were also screened for additional relevant content. Only studies reporting clinical trials were considered for the review.

### 2.1 The role of diet

**FIGURE 2:**
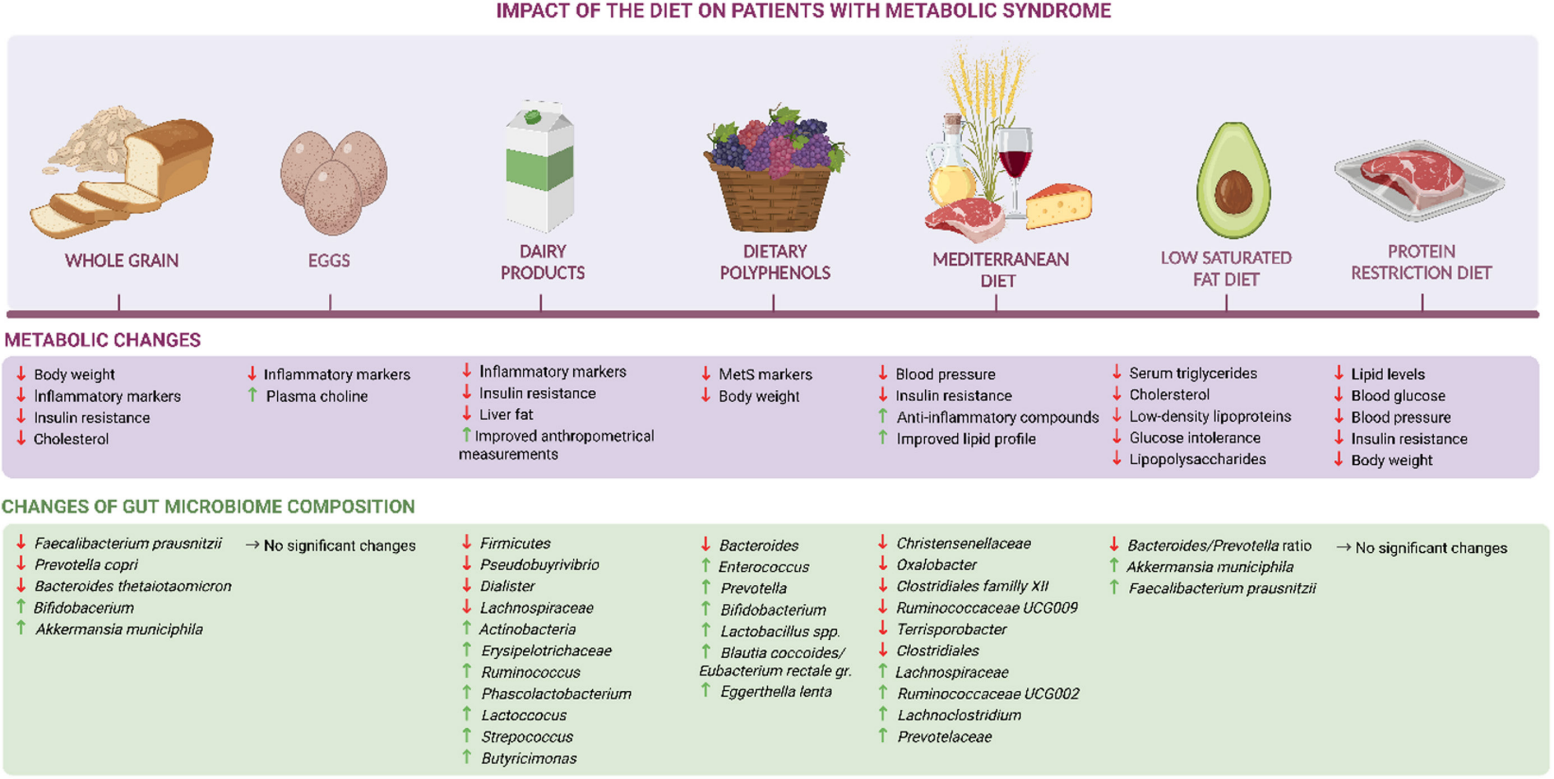
Summary of the impact of diet on patients with metabolic disease focussing on changes in gut microbiome composition.

Changes in the diet can alter energy balance, provide various nutrients and modulate the gut microbiome, which can have different effects on the development of diseases, including obesity and MetS. More than a decade ago, this was proven by Vijay‐Kumar et al.,^11^ who provided evidence for the direct relationship between changes in the composition of the gut microbiota of mice and the development of the MetS. The role of certain diets or food ingredients on MetS in humans and the human gut microbiome remains unclear; thus, it is necessary to study it in more detail. The included studies with their clinical endpoints are summarized in Table 1 and Figure 2.

**TABLE 1:** Clinical studies on diet and the gut microbiome in patients with MetS.

Study	No. of clinical trial	Population/number of participants	Duration	Intervention	Primary endpoint	Result metabolic	Result microbiome	Adverse events
Roager, 2017^20^	Registered at clinicaltrials.gov NCT01731366	50 volunteers at risk of developing MetS	Two 8‐week intervention periods separated by ≥6 weeks washout period	Whole‐grain diet	Replacing refined grains with whole grains improves insulin sensitivity and alters the gut Microbiome	Whole‐grain diet did not improve insulin sensitivity but reduced body weight. It reduced fasting circulating levels of low‐grade inflammatory markers without affecting intestinal permeability	Relative to the refined grain diet, the whole‐grain diet did not result in significant changes in the faecal bacterial species diversity or richness of the microbial community	Not reported
Malin, 2018^21^	Registered at clinicaltrials.gov NCT01411540	14 moderately obese volunteers (34.0 ± 1.1 kg/m^2^)	8‐week intervention with an 8–10 week washout period between diets	Whole‐grain diet	Whole‐grain diet reduces insulin resistance and improves glucose use in individuals at risk for type 2 diabetes compared with an isocaloric‐matched refined‐grain diet	Post‐prandial glucose tolerance, peripheral insulin sensitivity and metabolic flexibility improvements were greater after whole‐grain compared with the refined grain diet	Not reported	Not reported
Eriksen, 2020^58^	Registered at clinicaltrials.gov NCT02987595	49 volunteers with signs of MetS	8‐week intervention period	Whole‐grain diet	Impact of whole‐grain rye, alone and with lignan supplements and whole‐grain wheat diets on glucose tolerance, other cardiometabolic outcomes, enterolignans and microbiota composition	Plasma glucose and insulin concentration profiles did not differ between or within diets. No differences were observed between intervention diets for BMI, waist circumference, triglycerides and systolic or diastolic blood pressure	Whole‐grain rye resulted in a higher abundance of Bifidobacterium compared with baseline and a lower abundance of Clostridium genus compared with whole‐grain wheat	Not reported
Velikonja, 2018^22^	Registered at clinicaltrials.gov NCT02041104	43 volunteers with high risk for MetS or with diagnosed MetS	4‐week intervention period	Barley β‐glucans	Changes in gut microbiota composition, production of SCFA and improvement of metabolic status in patients with MetS	Total plasma cholesterol decreased in the test group. Short‐chain fatty acids composition in faeces significantly changed with an increase of propionic acid in the test group and with a decrease of acetic acid in the control group	Decrease in microbial diversity and richness in the test group. The pre‐intervention gut microbiota composition showed a higher abundance of health‐associated *Bifidobacterium* spp. and *Akkermansia municiphila* within the cholesterol‐responsive group	Not reported
Thomas, 2022^32^	Registered at clinicaltrials.gov NCT03877003	23 volunteers with MetS	13‐week trial: 2 weeks wash‐out, 4 weeks intervention, 3 weeks wash‐out, 4 weeks intervention	Eggs or choline bitartrate supplement	Effects of choline on the gut microbiome in MetS patients	The number of one carbon metabolites in plasma increased, with no change in methionine and trimethylamine oxide	Gut microbiota profiles of 23 MetS patients showed no significant difference in α‐ or β‐diversity	Not reported. Further studies with extended periods or different populations need to be conducted
Liu, 2021^33^	Registered at www.chictr.org.cn as ChiCTR2100046956	9 volunteers with a low risk of developing metabolic disease	2‐week dietary intervention	Eggs or choline bitartrate supplement	Change in vascular function and microbiota composition	No effects	No effects	Not reported
Bellicki‐Koyu, 2019^37^	Registered at clinicaltrials.gov NCT03966846	22 volunteers with MetS	12‐week intervention period	Kefir	Changes in lipid, glycemic profile and in gut microbiota	Improvements in anthropometrical measurements, lipid profile, glycemic status, and inflammation	The relative abundance of *Actinobacteria* phylum was increased in the kefir group	Not reported
Chen, 2019^37^	Registered at www.chictr.org.cn as ChiCTR‐IPR‐15006801	92 obese volunteers with WC ≥ 90 cm and BMI ≥28 kg/m^2^	24‐week intervention period	Yogurt	Improvement in fast blood glucose, insulin and hepatic lipid contents	Improved insulin resistance and reduced liver fat	Lower abundance of the *Firmicutes* phylum, *Pseudobutyrivibrio* and *Dialister* genera in the yogurt group and a higher abundance of *Erysipelotrichaceae* family, *Ruminococcus* genus, an increase in *Phascolarctobacterium* genus	Not reported
Moreno‐Indias, 2016^45^	—	10 obese volunteers that met the criteria for the MetS and 10 healthy subjects (control group)	4 periods: a washout period of 2 weeks, two intervention periods of 30 days, a washout period of 15 days	Red wine	Improvement in the risk factors for the MetS in obese patients	Reduction in the metabolic syndrome risk markers	In the MetS patients significantly increased intestinal barrier protectors and butyrate‐producing bacteria	Not reported
Galie, 2021^51^	Registered at isrctn.com ISRCTN88780852	44 volunteers with MetS	2‐month dietary intervention trial with a 1‐month wash‐out period	Mediterranean diet	The effect of the Mediterranean diet on circulating metabolites in patients with MetS	Mediterranean diet was associated with changes in the plasma metabolome that were associated with insulin resistance improvements	An uncultured genus of *Lachnospiraceae*, *Ruminococcaceae UCG002*, *Lachnoclostridium* and some genera from *Prevotellaceae* family was positively correlated with changes of the metabolome. Uncultured genus from *Christensenellaceae* family, *Oxalobacter*, *Clostridiales family XII*, *Ruminococacceae UCG009*, *Terrisporobacter* and a genus from *Clostridiales* family, was negatively correlated with changes of metabolome	Not reported
Haas, 2022^52^	Registered at *clinical trials.gov* NCT03232099	42 men with diagnosed coronary artery disease	3‐week red wine consumption (250 mL/d, 5 d/week) with an equal period of alcohol abstention, both preceded by a 2‐week washout period	Red wine	Changes in gut Microbiome and metabolites composition	Not reported	Difference in beta diversity and predominance of *Parasutterella*, *Ruminococcaceae*, several *Bacteroides* species, and *Prevotella*.	Not reported
Ferraz‐Bannitz, 2022^56^	Registered at ensaiosclinicos.gov.br RBR‐3HKNRW	21 volunteers with diagnosed MetS	27‐day trial, with follow‐up after ~30 days after hospital discharge	Isocaloric dietary protein restriction diet	Isocaloric dietary protein restriction is sufficient to confer the beneficial effects of dietary restriction in patients with MetS	Weight loss, reductions in blood glucose, lipid levels and blood pressure, and improved insulin sensitivity	Gut microbiome diversity was not affected by the interventions	Not reported
Guevara‐Cruz, 2019^59^	Registered at clinicaltrials.gov NCT03611140	1032 volunteers with overweight and obesity and 542 of them MetS was confirmed	2‐month intervention period for the 1st part of the study and a 2‐week intervention period for the 2nd part of the study	Low‐saturated‐fat diet	Low‐saturated‐fat diet positively affects biochemical parameters, gut microbiota and metabolic endotoxemia in MetS patients	After 15 days there was a 24% reduction in serum triglycerides; and after a 75‐day lifestyle intervention, MetS was reduced by 44.8%, with a reduction in low‐density lipoprotein cholesterol, small low‐density lipoprotein particles, glucose intolerance, lipopolysaccharide, and branched‐chain amino acid	Decrease in the Prevotella/*Bacteroides* ratio and an increase in the abundance of *Akkermansia muciniphila* and *Faecalibacterium prausnitzii* after lifestyle intervention	Not reported

Abbreviations: MetS, metabolic syndrome; WC, waist circumference.

### 2.2 Whole‐grain diet

A whole grain is any grain that contains the endosperm, germ and bran, as opposed to refined grains that retain only the endosperm. Whole‐grain products are metabolized into short‐chain fatty acids,^12^ which are involved in glucose and lipid metabolism,^13^, ^14^ immune homeostasis^15^ and intestinal permeability.^16^ As part of an overall healthy diet, consuming whole grains is associated with a lower risk of several diseases, including type 2 diabetes and cardiovascular diseases.^15^, ^17^ Insufficient consumption of whole grains has been listed as a contributing factor to a higher risk of disability‐adjusted life years in worldwide studies.^18^ Moreover, the consumption of whole‐grain products, nuts and seeds may have the greatest health benefits of all the dietary factors studied, including fruit and vegetable consumption.^19^

A randomized, controlled cross‐over clinical trial, which aimed to investigate the effects of a whole‐grain diet on insulin sensitivity, metabolic health and its impact on the gut microbiome compared with refined grain, did not show any significant effect of a whole‐grain diet on glucose homeostasis and did not induce major changes in the diversity or richness of the gut microbiome.^20^ Analysing distinct species, the abundance of four distinct strains of *Faecalibacterium prausnitzii* and one strain of *Prevotella copri* increased after adhering to a whole‐grain diet and decreased in a refined‐grain diet, whereas *Bacteroides thetaiotaomicron* showed opposite tendencies. However, the whole‐grain diet showed a positive impact on overall metabolic health as body weight and serum inflammatory markers were reduced in the study group.^20^

Another randomized, double‐blind, controlled crossover clinical study similarly aimed to determine how a whole‐grain diet affects glucose tolerance and insulin resistance, compared with a refined grain diet. As opposed to the previously described study, these parameters improved with adherence to the whole‐grain diet, suggesting its potential positive effects on diabetes.^21^ However, this study was conducted merely on 14 moderately obese adults at risk for diabetes, not including patients with clinically confirmed MetS. Moreover, in this study, the authors did not analyse the diet's impact on the changes in gut microbiome composition. Although the intervention time in both studies was 8 weeks, the differences in the results could be due to the chosen diet strategies.^21^

Barley beta‐glucans, which form highly viscous solutions in the gut, thus extending the transit time of nutrients through the gut, may modify gut microbiota composition and improve overall metabolic health in MetS patients.^22^, ^23^ It has been shown that a diet enriched with barley beta‐glucans improved markers of MetS, as cholesterol levels were reduced in the test group. The analysis of gut microbiota composition, however, showed that bacterial diversity and richness both decreased after the dietary intervention with barley beta‐glucans.^22^ Loss of diversity is usually related to gastrointestinal or liver diseases.^24^ However, greater gut microbial richness or diversity is not an absolute sign of a healthy gut, as could be observed in several diseases;^25^, ^26^ thus, further studies on the impact of barley beta‐glucans on MetS are needed.

### 2.3 Eggs

Eggs, being an essential component of the human diet, play an important role in human health. Egg yolks are known to contain highly available forms of antioxidants, such as choline or lutein carotenoids and zeaxanthin, which can protect against the development of metabolic diseases. This protection is thought to be due to an increased mRNA expression of antioxidant enzymes and a decreased efficiency of pro‐inflammatory cytokines.^27^ Interestingly, many studies show that the consumption of egg yolks rich in cholesterol does not increase the risk for cardiovascular diseases.^28^, ^29^, ^30^ The effects of eggs on the gut microbiome remain largely unknown.

The effect of two sources of choline—whole eggs and a choline supplement (choline bitartrate)—on plasma lipids, glucose, insulin resistance and inflammatory biomarkers were evaluated. An increase in plasma choline in individuals with MetS, who consumed either three eggs per day or a choline supplement was observed. Interestingly, decreased inflammation was detected only after egg intake, which could be due to the antioxidants present in eggs.^31^ A similar study aimed to further explore the effects of choline provided by eggs or choline supplements on the gut microbiota in subjects with MetS. However, although blood plasma values of choline were increased, diet intervention had no significant effects on microbiome diversity parameters or relative taxa abundances, possibly due to the short intervention period.^32^ Longer intervention times could be needed to obtain significant changes in the gut microbiome. Similarly, no relevant changes in the taxonomic composition of the gut microbiome were reported after a 2‐week intervention period with two eggs per day.^33^

### 2.4 Dairy products

Dairy products are known as a source of multiple essential vitamins, minerals and high‐quality protein. In addition, additional health benefits can be obtained after milk fermentation. Fermented dairy products are among the most effective sources of probiotics.^34^ Kefir is a fermented milk product, which has a very specific combination of bacteria and yeasts, present in grains.^35^ *Lactobacillus*, *Lactococcus*, *Streptococcus leuconostoc* and acetic acid bacteria are the most common bacteria, and *Saccharomyces*, *Kluyveromyces* and *Candida* are commonly found yeasts in kefir grains.^36^ Knowing the benefits of kefir to overall health, it was aimed to study its potential effect on gut microbiota in MetS patients. Knowing the benefits of kefir to overall health, it was the aim of a clinical trial to study its potential effect on gut microbiota in MetS patients: The consumption of 180 mL of kefir per day in MetS patients resulted in a significant increase in the relative abundance of *Actinobacteria*. A strong positive correlation with systolic and a weak positive correlation with diastolic blood pressure and *Actinobacteria* abundance was observed compared with the consumption of unfermented milk in the control group.^37^

The benefits of fermented dairy products on changes in insulin resistance were investigated in a randomized, parallel and controlled clinical study where MetS patients with fatty liver disease consumed 220 g per day of yogurt or unfermented milk (in the control group). Yogurt had higher effects at ameliorating insulin resistance than unfermented milk and decreased the abundances of *Firmicutes* phylum, the *Clostridiales* order and *Blautia* and *Eubacterium ventriosum* group genera.^38^ Yogurt also decreased the *Erysipelotrichaceae* family and the *Ruminococcus* genus, which are genera associated with increased liver fat and increased *Phascolarctobacterium* genus but decreased *Pseudobutyrivibrio* and *Dialister* genera, which are associated with lipid metabolism, liver injury and insulin resistance.^38^, ^39^, ^40^ Bryndza is a traditional Eastern European cheese made from sheep milk, which is rich in butyric acid. The consumption of Bryndza, as part of a weight loss program, did not advance weight loss, BMI reduction or changes in the body composition. However, it increased the abundance of Lactococcus, Streptococcus, Phascolarctobacterium and Butyricimonas in the gut microbiome, while decreasing taxa from the Lachnospiraceae family, which include many potent butyrate producers.^41^

### 2.5 Dietary polyphenols

Polyphenols are naturally found in various plant parts, such as leaves, stems and flowers.^42^ According to a recent study, the average polyphenol intake for the general population was estimated to be 0.9 g perday, and the main sources were not only fruits and vegetables but also beverages, such as coffee, tea and red wine.^43^ The concern about the impact of the consumption of red wine on the gut microbiome composition is increasing. Significant increases in the abundance of *Enterococcus*, *Prevotella*, *Bacteroides*, *Bifidobacterium*, *Bacteroides uniformis*, *Eggerthella lenta* and the *Blautia coccoides*‐*Eubacterium rectale* group were observed, indicating potential positive effects of polyphenols in red wine that must be weighed against the well‐known negative effects of alcohol on the liver and metabolic health.^44^ In a small randomized, crossover‐controlled intervention study, red wine consumption was associated with a greater reduction in body weight and a significant decrease in *Bacteroides* as well as a significant increase in protectors of the gut mucosal barrier, such as *Bifidobacterium* spp. and *Lactobacillus* spp.^45^

### 2.6 Specialized diets

The Mediterranean diet (MedDiet) includes a high consumption of fruits, vegetables, whole‐grain foods, fish, nuts and olive oil. In addition, it is characterized by a low intake of red wine, meat and dairy products.^46^ MedDiet is rich in food primarily containing unsaturated fatty acids.^47^ It is proved that this diet has positive effects on lipid profile, glycemic control and blood pressure,^48^ as it provides anti‐inflammatory compounds.^49^ MedDiet can be used as a potential therapy for MetS to prevent excess adiposity and related inflammatory responses associated with obesity.^50^ The effects of MedDiet on insulin resistance and gut microbiome composition were studied in a crossover randomized clinical trial in MetS patients; MedDiet was associated with changes in the plasma metabolome that were associated with improvements of insulin resistance and correlated with the uncultured genus of *Lachnospiraceae*, *Ruminococcaceae UCG002*, *Lachnoclostridium,* and some genera from the *Prevotellaceae* family. Moreover, negative correlations with changes in phosphoethanolamine and taurine were found for these bacteria.^51^

While more research is needed to fully understand the mechanisms underlying MedDiet's impact on improving gut microbiome health, it is considered that the previously described polyphenols and other bioactive compounds found in red wine can have a positive impact. Red wine consumption was significantly associated with an increase in the relative abundance of *Bacteroides*, *Ruminococcaceae*, *Roseburia* and *Prevotella*. Interestingly, *Parasutterella* was the most prominent genus for differentiating the gut microbiota of the participants who consumed red wine.^52^ A previous study showed that *Parasutterella* supports interspecies metabolic interactions within the healthy gut ecosystem.^53^ A recent review investigated the potential benefits of theaflavins, found in red wine, on metabolic syndrome, with a focus on the gut microbiome. It was discussed that theaflavins have the potential to positively impact the gut microbiota by increasing the abundance of beneficial gut bacteria such as *Lachnoclostridium* and *Bifidobacterium*.^54^

A diet rich in proteins was shown to have a negative impact on health and is associated with increased mortality.^55^ In a randomized clinical trial, patients with MetS received caloric restriction or isocaloric dietary protein restriction; both interventions resulted in positive metabolic outcomes such as weight loss, improved insulin sensitivity, blood glucose, lipid and blood pressure without any significant changes in the microbiome, suggesting that dietary metabolic health may only be indirectly related to the microbiome.^56^ While a protein‐rich diet was thought to have negative health effects, studies have shown that it can be beneficial in terms of body composition and metabolism. Several studies have investigated the potential impact of plant‐based protein consumption on reducing cardio‐metabolic risk factors. A systematic review and meta‐analysis of 112 trials conducted on adults with and without hyperlipidemia found that plant protein intake as a substitute for animal protein resulted in reduced markers (low‐density lipoprotein cholesterol, non‐high‐density lipoprotein cholesterol and apolipoprotein B) of cardiovascular disease.^57^

### 2.7 Probiotics

‘Probiotics’ is a collective term for living organisms (usually bacteria or fungi) that can exert health benefits on the host if consumed in sufficient amounts.^60^ In fermented foods, probiotic bacteria have been a part of human nutrition for a long time but have been neglected in Western‐style diets, which are associated with the current obesity epidemic.^61^ Meta‐analyses show that supplementation with probiotics has been successfully used to improve classical traits of T2D, such as fasting plasma glucose, insulin concentration, insulin resistance, glycated haemoglobin (HbA1c)^62^, ^63^, ^64^ and improved lipid parameters.^65^ Furthermore, a slight improvement in body weight, BMI and fat percentage following a probiotic intervention has been observed in obese and overweight people.^66^ Accordingly, probiotics might be a valuable asset in the treatment of obesity, MetS and diabetes, but the available data are heterogenous in regard to study design, products, duration of intervention and outcome and is, therefore, inconclusive. Furthermore, the working mechanisms behind the observed effects and the involvement of the microbiome and microbiome modulation are still not understood. Table 2 summarizes the current body of literature focusing on probiotic microbiome modulation to ameliorate obesity, MetS or type 2 diabetes. Probiotics have been tested as single‐strain or multi‐strain/multi‐species formulations, sometimes with a prebiotic substance, resulting in a so‐called synbiotic.

**TABLE 2:** Clinical studies testing probiotics as microbiome modulators in obesity, MetS and diabetes.

Study	Population/number of participants	Duration	Intervention	Primary endpoint	Result metabolic	Result microbiome	Adverse events
Nagata et al. 2017^67^ UMIN000006958	12 obese children on diet and exercise therapy	24 weeks	*Lactobacillus casei* Shirota (LcS) (4 × 10^10^ CFU) No control group	Weight loss, hyperlipidemia and blood sugar reduction	Weight ↓ HDL and acetic acid ↑	Bifidobacteria and *L. casei* ↑	Not reported
Sato et al. 2017^68^ UMIN000018246	70 patients with type 2 diabetes	16 weeks	Probiotic: *Lactobacillus casei* strain Shirota‐fermented milk Control: no intervention	Gut microbiota, detection rates and bacterial blood counts	Organic acids and total count of bacteria in blood ↓ faecal pH, hs‐CRP and T‐CHO ↑	Probiotics: Lactobacillus and *L. casei*, *L. reuteri* and *L. gasseri* counts, *C. coccoides* group and the *C. leptum* subgroup ↑ Control: Bifidobacterium, Atopobium cluster, total Lactobacillus, and *L. fermentum* ↑ Prevotella ↓	Not reported
Stadlbauer et al. 2015^69^ NCT01182844	28 subjects with metabolic syndrome	12 weeks	*Lactobacillus casei* Shirota (LcS) Control: no intervention	Post‐hoc analysis of previous trial (PMID: 22872030)	No effect on gut barrier, serum bile acids	Parabacteroides ↑	Not reported
Tenorio‐Jimenez et al. 2019^71^ NCT02972567	53 adult patients newly diagnosed with MetS	12 weeks	*L. reuteri* V3401 (5 × 10^9^ CFU) Control: Placebo	Improving MetS components	Il‐6, sVCAM and insulin levels ↓	Verrucomicrobia/Akkermansia muciniphila ↑	Not reported
Hsieh et al. 2020^72^ NCT02274272	74 people with treatment naive T2DM	24 weeks	Groups: 1. live *L*. *reuteri* ADR‐1 (4 × 10^9^ CFU) 2. heat‐killed *L*. *reuteri* ADR‐3 (2 × 10^10^ CFU) 3. Placebo	Change in blood sugar	ADR‐1 group: HbA1c ↓ ADR‐3 group: systolic blood pressure, mean arterial pressure and IL‐1b ↓	ADR‐1 group: *L. reuteri* ↑ ADR‐3 group: *L. reuteri* and Bifidobacterium ↑	Safe, without any adverse effects
Sohn et al., 2022^74^ KCT0003944	81 adults with a body mass index of 25–30 kg/m^2^	12 weeks	*Probiotic: Lactobacillus plantarum* K50 (4 × 10^9^ CFU) Control: Placebo	Any change in the subjects' body fat mass	Total cholesterol and triglyceride levels ↓	*Actinobacteria* ↓ *L. plantarum, Enterococcus/Enterococcus hirae* ↑	Only mild adverse events reported, no significant differences between groups and no relevant relation to the study medication
Oh, et al 2021^75^ KCT0005652	40 prediabetic adults	8 weeks	*L. plantarum* HAC01 Control: Placebo	2 h‐PPG improvement	2 h‐PPG and HbA1c ↓	No change in faecal microbiome	No severe adverse events, adverse events similar in both groups
Rahayu et al. 2021^76^ INA‐2A8RG4R	60 overweight patients	12 weeks	*L. plantarum Dad‐13* (2 × 10^9^ CFU) Control: Placebo	Not stated	Body weight and BMI ↓	Firmicutes ↓ Bacteroidetes ↑	Not reported
Larsen et al. 2013 (PMID: 23510724) NCT01020617	51 obese adolescents	12 weeks	*Probiotic: L*. *salivarius* Ls‐33 ATCC SD5208 (10^10^ CFU) Control: Placebo	Post hoc analysis of previously reported trial	No change in SCFA, metabolic changes not reported	Probiotic recovered, no significant shift in the overall composition	Not reported
Crovesy et al. 2021 (PMID: 33565558) NCT02505854	51 adult women with obesity during low‐caloric diet	8 weeks	Probiotics: Bifidobacterium lactis UBBLa‐70 Synbiotics: Bifidobacterium lactis UBBLa‐70 and fructooligosaccharide Control: Placebo	Weight loss	Synbiotic group: Pyruvate and alanine ↑ Citrate, isoleucine and BCAA↓ Probiotic group: Serum lipids ↓ No differences in weight loss	Changes in gut microbiome not reported, correlations between microbiome and metabolites	Not reported
Hibberd et al, 2019 (PMID: 30525950) NCT01978691	134 overweight adults	24 weeks	Groups: 1. Litesse® Ultra™ polydextrose (12 g) + Bifidobacterium animalis subsp. lactis 420™ (B420) (10^10^ CFU) 2. Bifidobacterium animalis subsp. lactis 420™ (B420) (10^10^ CFU) 3. Litesse® Ultra™ polydextrose (12 g) 4. Placebo	Posthoc analysis of MetSProb Study	Not reported	Changes mainly observed in the prebiotic and synbiotic group, minimal changes in the probiotic group	Not reported
Krumbeck et al. 2018 (PMID: 29954454) NCT02355210	114 oberweight adults	3 weeks	Syn‐ and Probiotics: B. adolescentis IVS‐1 or B. animalis BB‐12 with or without 6.9 g of Vivinal Control: Placebo	Gut permeability	No change in gut permeability BB‐12 + GOS: HDL‐cholesterol ↑ BB‐12 group: HDL‐cholesterol ↓	Probiotics with and without GOS: increase in probiotic strans and related taxa IVS‐1 + GOS: Roseburia ↓ GOS: Lachnobacterium ↓, Bifidobacteria and Actinobacteria ↑	Generally well tolerated with minimal reported side effects
Gutierrez‐Repiso et al. 2019 (PMID: 31298466) NCT03530501	Adults with BMI >30 kg/m^2^ entering a weight loss programm	16 weeks	1. Group: 2 months very low‐caloric ketogenic diet + synbiotic 1 and 2 month low‐caloric diet + synbiotic 2 2. Group: 2 months very low caloric ketogenic diet + placebo and 2 months low‐caloric diet + synbiotic 2 3. group: 2 months very low‐caloric ketogenic diet + placebo and 2 months low‐caloric diet + placebo Synbiotic 1: Bifidobacterium lactis, L. rhamnosus, Bifidobacterium longum ES1 and prebiotic fibre Synbiotic 2: Bifidobacterium animalis subsp. lactis and prebiotic fibre	Composition and diversity of gut microbiota	Placebo‐synbiotic2: weight loss ↑, GGT levels ↓	Synbiotic1‐synbiotic2: Prophyromonadaceae, Christenellaceae, Parabacteroides, Bulleidia and Odoribacter ↑ Placebo‐synbiotic2: Prophyromonadaceae, Christenellaceae, Parabacteroides, Lachnospira ↑ Placebo: 02d06 ↑	Not reported
Pellegrini et al. 2020 (PMID: 32234652) Registration number not given	34 female breast cancer survivors	8 weeks	Probiotic: B. longum BB536 (4 × 10^9^ CFU) and L. rhamnosus HN001 (1 × 10^9^ CFU) Groups: 1. Mediterranean diet + probiotics 2. Mediterranean diet	Unclear	Waist circumference, waist‐to‐hip ratio and insulin levels ↓	Alpha diversity ↑ Eubacterium and L‐Ruminococcus ↑ Bacteroides and Butyricicoccus ↓	Not reported
Solito et al. 2021 (PMID: 34229263) NCT03261466	101 obese children and adolescents during dietary weight loss treatment	8 weeks	*Bifidobacterium breve* BR03 (DSM 16604) and *B. breve* B632 (DSM 24706) (2 × 10^9^ CFU) Control: Placebo	Improvement in glucose metabolism	Waist circumference ↓, BMISDS ↓, fasting insulin ↓, ALT↓	Changes similar to placebo group	No adverse events were reported in any part of the study
Mo et al 2022 (PMID: 35745214) KCT0007117	72 otherwise healthy obese and overweight patients	12 weeks	Probiotic: *L. curvatus* HY7601 (5 × 10^9^ CFUs), *L. plantarum* KY1032 (5 × 109 CFUs) Control: Placebo	Body weight, BMI, waist circumference and hip circumference	Body weight and BMI ↓ Waist circumference ↓ Body fat mass ↓ Lean body mass ↓ Visceral fat area ↓ Leptin ↓ Adiponectin ↑	Probiotics group: Bifidobateriaceae and Akkermansiaceae ↑ B. adolescentis, B. longum and A. muciniphila ↑ Oscillospiraceae, Selenomonadaceae, and Prevotellaceae ↓ Collinsella aerofaciens, *Faecalibacterium prausnitzii* and *Prevotella copri* ↓ placebo group Selenomonadaceae, Prevotellaceae, Coriobacteriaceae, and Eggerthellaceae ↑ Bacteroidaceae ↓ both groups: Alpha diversity ↓	Not reported
Jones et al. 2018 (PMID: 29493105) NCT03115385	19 obese Latino adolescents	16 weeks	Probiotics: VSL#3 (Streptococcus thermophilus, Bifidobacterium breve, Bifidobacterium longum subsp. longum, Bifidobacterium longum subsp. infantis, Lactobacillus acidophilus, Lactobacillus plantarum, *Lactobacillus paracasei* and Lactobacillus delbrueckii subsp. bulgarius) Control: Placebo	Gut microbiota and/or gut hormones	Increases in total adiposity (%) and trunk adiposity (%)	No effect	No severe adverse events during the intervention
Lee et al. 2013 (PMID: 24411490) KCT0000386	50 females with BMI >25 kg/m^2^	8 weeks	Probiotics: Streptococcus thermophiles (KCTC 11870BP), Lactobacillus plantarum (KCTC 10782BP), Lactobacillus acidophilus (KCTC 11906BP), Lactobacillus rhamnosus (KCTC 12202BP), Bifidobacterium lactis (KCTC 11904BP), Bifidobacterium longum (KCTC 12200BP), and Bifidobacterium breve (KCTC 12201BP) (5 × 10^9^ CFU) Groups: 1. Bofutsushosan (BTS) + probiotics 2. BTS alone	Difference in weight and gut permeability	HDL ↑ no other changes	B. breve, B. lactis and L. rhamnosus ↑ Gram negative bacteria ↑	Not reported
Palacios et al. 2020 (PMID: 32660025) ACTRN12613001378718	60 adults with prediabetes and early T2DM	12 weeks	*Lactobacillus plantarum* Lp‐115 (6 × 10^9^ CFU), *Lactobacillus bulgaricus* Lb‐64 (3 × 10^9^ CFU), *Lactobacillus gasseri* Lg‐36 (18 × 10^9^ CFU), *Bifidobacterium breve* Bb‐03 (7.5 × 10^9^ CFU), *Bifidobacterium animalis sbsp. lactis* Bi‐07 (8 × 10^9^ CFU), *Bifidobacterium bifidum* Bb‐06 (7 × 10^9^ CFU), *Streptococcus thermophilus* St‐21 (450 × 10^6^ CFU) and *Saccharomyces boulardii* DBVPG 6763 (45 × 10^6^ CFU) Control: Placebo	Fasting plasma glucose	No changes in metabolic parameters	Probiotics: Bifidobacteria recovered, Bacteroides caccae, Bacteroidales bacterium ph 8, Akkermansia muciniphila, Clostridium hathewayi ↑ *Prevotella copri*, Flavonifractor plautii ↓ Placebo: Bacteroides faecis, finegoldii, salyersiae and thetaiotaomicron, Parabacteroides merdeae and Bilophila wadsworthia ↑ Desulfovibrio desulfuricans ↓	Four mild lower limb infections in probiotic group, one urinary tract infection in the placebo group, no serious adverse events, no difference in GI symptoms between groups
Gomes et al. 2019 (PMID: 31250099) U1111‐1137‐4566	60 overweight or obese women	8 weeks	*Lactobacillus acidophilus* LA‐14, *Lactobacillus casei* LC‐11, *Lactococcus lactis* LL‐23, *Bifidobacterium bifidum* BB‐06 and *Bifidobacterium lactis* BL‐4 (2 × 10^10^ CFU) Control: Placebo	Post hoc analysis of previously reported trial	Not reported	Probiotics: Firmicutes ↑ Collinsella, Turicibacter, Clostridiaceae, Dorea, Peptostreptococcaceae, Tissierellaceae and Catenibacterium ↑ Bacteroides and Fusobacteria↓ Prevotella, Odoribacter, Bacteroidales and Fusobacterium ↓ Control: Bacteroidetes and Verrucomicrobia ↓ Collinsella, Bacteroides and Anaerotruncus ↓ Gammaproteobacteria and Akkermansia ↑	Not reported
Firouzi, et al. 2016 (PMID: 26988693) NCT01752803	136 patients with T2D	12 weeks	Lactobacillus acidophilus, *Lactobacillus casei*, Lactobacillus lactis, Bifidobacterium bifidum, Bifidobacterium longum and Bifidobacterium infantis (10^10^ CFU each) Control: Placebo	HbA1c	HbA1c, fasting insulin and HOMA‐IR ↓	Lactobacillus and Bifidobacteria count ↓	Minor gastric disturbances while two unexpected events were observed in the probiotic group (sexual impotency and carbuncle), unlikely due to the intervention
Kaczmarczyk et al 2022 (PMID: 35360106) NCT03100162	56 postmenopausal obese women	12 weeks	Probiotic: Bifidobacterium bifidum W23, Bifidobacterium lactis W51, Bifidobacterium lactis W52, Lactobacillus acidophilus W37, Levilactobacillus brevis W63, *Lacticaseibacillus casei* W56, *Ligilactobacillus salivarius* W24, Lactococcus lactis W19 and Lactococcus lactis W58 1. test group: 1 × 1010 colony forming units (CFU) per day 2. test group: 2.5 × 109 colony forming units (CFU) per day control: placebo	Post hoc analysis of previously reported trial (PMID: 29914095)	Previously reported improvement in LPS, waist circumference, fat mass, subcutaneaous fat, uric acid, total cholesterol, triglycerides, LDL, glucose insulin, HOMA‐IR (PMID: 29914095)	Bray–Curtis dissimilarity decreased in high‐dose probiotic group unchanged: alpha diversity, bacterial abundances and function over time	Not reported
Horvath et al, 2019 (PMID: 31729622) NCT02469558	49 diabesity patients (T2D + obesity)	24 weeks	*Synbiotics: B. bifidum W23*, *B. lactis W51*, *B. lactis W52, L. acidophilus W37*, *L. casei W56*, *L. brevis W63*, *L. salivarius W24*, *Lc. lactis W58* and *Lc. lactis W19 (1.5 × 10* ^*10*^ *CFU) + Galacto‐oligosaccharides P11 (GOS) and Fructo‐oligosaccharides P6 (FOS) Control: Placebo*	Glucose metabolism	No significant change in glucose metabolism Hip circumference and lipoprotein (a) (LPA) ↓ Quality of life (SF36) ↑	Increase in L. brevis (Probiotic strain), no change in microbiome	7 serious adverse events not related to the intervention (Biliary colic, decompensated heart failure, invasive gastroenteritis, hospitalization for pain management and impaired renal function, cerebral tumour, Helicobacter pylori associated gastritis, hospitalization for management of arterial hypertension)
Jancy et al. 2020, (PMID:33326198) Registration number not given	60 adult persons with BMI ≥ 25 kg/m^2^	12 weeks	Synbiotics: Bifidobacterium lactis W51(NIZO 3680)/W52 (NIZO 3882) (2.8 × 10^8^), Lactobacillus acidophilus W22 (NIZO 3674) (1.2 × 10^8^), lactobacillus paracasei W20 (NIZO 3672) (0.9 × 10^8^), Lactobacillus plantarum W21 (NIZO 3673) (1.1 × 10^8^), Lactobacullus salivarius W24 (NIZO 3675) (0.9 × 10^8^), Lactococcus lactis 19 (NIZO 3671) (1.1 × 10^8^), Fructooligosaccharides (FOS) 96 mg, Inulin 110.4 mg) Control: Placebo	Not clearly stated	No change in body mass reduction zonulin ↓	Lactobacilli and total bacteria ↑ Proteolytic bacteria ↓	Not reported
Sergeev et al. 2020 (PMID: 31952249) NCT03123510	20 patients starting weight loss programm	12 weeks	Synbiotics: Lactobacillus acidophilus DDS‐1, Bifidobacterium lactis UABla‐12, Bifidobacterium longum UABl‐14, and Bifidobacterium bifidum UABb‐10 (15 × 109 CFU) + 5.5 g trans‐galactooligosaccharide (GOS) Groups: 1. Weight loss programm + synbiotic 2. Weight loss programm alone	Changes in gut microbiota	HbA1c ↓	*Cyanobacteria, Euryarchaeota, Fusobacteria,* and *Lentisphaerae, Ruminococcus, Bifidobacterium, Sutterella, Tyzzerella, Eisenbergiella, Eubacterium, Eggerthella, Methanobrevibacter, Lachnospiraceae, Edwardsiella, Lactobacillus, Allobaculum, Enterococcus, Hydrogenoanaerobacterium, Coprococcus, and Butyricimonas* ↑ *Ruminococcaceae, Prevotella, Gardnerella, Turicibacter, and Megasphaera* ↓	Not reported
Kanazawa, et al. 2021 (PMID: 33567701) UMIN000032057	88 obese T2DM patients	24 weeks	Synbiotics: *Lacticaseibacillus paracasei* YIT 9029 (strain Shirota: LcS) (3 × 10^8^ CFU), *Bifidobacterium breve* YIT 12272 (BbrY) (3 × 108 CFU), and 7.5 g GOS Control: no intervention	Change in the level of interleukin‐6 (IL‐6)	Fasting blood glucose and HbA1c after 12 weeks ↑, no change after 24 weeks lactic acid after 12 weeks ↑	Snybiotics after 12 weeks: Bifidobacterium, Lactobacillus, Lacticaseibacillus and Limosilactobacillus↑ Actinobacteriota ↑, Bacteroidota ↓ alpha diversity ↓ Bifidobacteriaceae ↑ Bacteroidaceae and Marinifilaceae ↓ Snybiotics after 24 weeks: Bifidobacterium, Prevotella, Lactococcus, Lactobacillus and Lacticaseibacillus ↑ Akkermansia ↓ Actinobacteriota ↑ Bacteroidota, Proteobacteria and Fusobacteriota ↓ Bifidobacteriaceae and Veillonellaceae ↑ Bacteroidaceae, Fusobacteria and Monoglobaceae ↓	No severe advere events, gastrointestinal symptoms in synbiotic group included diarrhoea, flatulence, soft stool and vomiting
Kassaian et al. 2020 (PMID: 32615392) registered at clinicaltrials.gov, no number given	120 patients with prediabetes	24 weeks	Probiotics: Lactobacillus acidophilus, Bifidobacterium lactis, B. bifidum, and Bifidobacterium longum; (1.5 × 10^9^ CFU each) Synbiotics: inulin + probiotics Control: Placebo	Unclear	Not reported	Probiotics: Bacteroides fragilis to *E. coli* ratio ↑ Firmicutes to Bacteroidetes repressive ↓	No difference in serious adverse events between groups
Zhang et al. 2020 (PMID: 33024120) NCT02861261 PREMOTE study	409 newly diagnosed T2D patients	12 weeks	Probiotic: Bifidobacterium longum CGMCC No. 2107, Bifidobacterium breve CGMCC No. 6402, Lactococcus gasseri CGMCC No. 10758, Lactobacillus rhamnosus CNCM I‐4474, Lactobacillus salivariusCGMCC No. 6403, Lactobacillus crispatus CGMCC No. 6406, Lactobacillus plantarum CGMCC No. 1258; Lactobacillus fermentum CGMCC No. 6407 and Lactobacillus caseiCNCM I‐4458 Groups: 1. Berberine + probiotics 2. Probiotics 3. Berberine 4. Placebo	Glycated haemoglobin	Berberine and berberine + probiotics but not probiotics alone reduced HbA1c and other metabolic parameters	Most changes in the microbiome introduced by berberine, independently of probiotics	Gastrointestinal adverse events in berberine groups
Wang et al., 2021 (PMID:34923903) NCT02861261	365 drug‐naive, newly diagnosed T2D patients	12 weeks	Probiotic: Bifidobacterium longum CGMCC No. 2107, Bifidobacterium breve CGMCC No. 6402, Lactococcus gasseri CGMCC No. 10758, Lactobacillus rhamnosus CNCM I‐4474, Lactobacillus salivariusCGMCC No. 6403, Lactobacillus crispatus CGMCC No. 6406, Lactobacillus plantarum CGMCC No. 1258; Lactobacillus fermentum CGMCC No. 6407 and Lactobacillus caseiCNCM I‐4458 Groups: 1. Berberine + probiotics 2. Probiotics 3. Berberine 4. Placebo	Post‐hoc analysis from the PREMOTE study – focus on postprandial lipidemia and microbiome	Synergistic effects on postprandial cholesterol, LDL and lipidomic metabolites; less or no effect of berberine and probiotics alone.	Most changes in the gut microbiome were found only in Prob + BBR treatment, less in berberine and probiotics group alone	Not reported
Hric et al. 2021, PMID: 34064069 Registration number not given	22 women in weight loss programm	30 days	Probiotic: Bryndza cheese (30 g) Groups: 1. weight loss programm + cheese 2. weight loss programm alone	Not clearly stated	No differences between groups	Lachnosiraceae ↓ Lactobacillales and Streptococcaceae ↑ Lactococcus, Streptococcus, Phascolarctobacterium and Butyricimonas ↑	Not reported

### 2.8 Single‐strain probiotics

*Lactobacillus casei* Shirota (LcS) is commonly available as a single‐strain probiotic milk drink. This milk drink given to obese children for 24 weeks as part of a diet and exercise therapy improved weight loss and increased HDL and acetic acid levels compared with an untreated control group. The intervention increased Bifidobacteria and *L. casei* counts in the gut microbiome of the treated children.^67^ These effects stand in direct contrast to the effects of LcS on Japanese adults with T2D. Although Lactobacillus abundance including *L. reuteri* increased in the microbiome, Bifidobacteria and HDL increased only in the control group. While patients treated with LcS showed a decreased count of bacteria in the bloodstream, organic acids decreased in the gut and hs‐CRP increased.^68^ The increase in hs‐CRP was also previously reported in a European cohort of MetS patients following a 12‐week LcS intervention, while neither changes in gut permeability or serum bile acids nor changes in the microbiome other than an increase in Parabacteroides were observed.^69^, ^70^

Lactobacillus reuteri V3401 in a single‐strain product reduced IL‐6, sVCAM and insulin levels in newly diagnosed MetS patients after 12 weeks of intervention, while it increasing the abundance of Verrucomicrobia/Akkermansia muciniphila.^71^ *L. reuteri* ADR‐1 decreased HbA1c and heat‐killed *L. reuteri* ADR‐3 decreased blood pressure, mean arterial pressure and IL‐1b and increased the abundance of Bifidobacteria in treatment‐naive T2D patients. Both groups showed an enrichment of the microbiome with the ingested probiotic but no other effects on the microbiome.^72^ Lactobacillus plantarum K50, a probiotic isolated from Kimchi, decreased total cholesterol and triglyceride levels in obese adults after a 12‐week intervention but had no effect on body weight or composition. The intervention increased the *L. plantarum*, the genus Enterococcus and Enterococcus hirae abundance, a potentially beneficial bacterium^73^ and decreased the abundance of Actinobacteria.^74^ *L. plantarum* HAC01 reduced 2‐h postprandial glucose (2 h‐PPG) and HbA1c levels in prediabetic adults without modulating the microbiome.^75^ *L. plantarum* Dad‐13 led to a decrease in body weight and BMI in obese adults, while decreasing Firmicutes and increasing Bacteroidetes in the microbiome.^76^ An intervention with *L. salivarius* Ls‐33 in obese adolescents showed no effect on metabolic parameters nor on the microbiome.^77^

Bifidobacterium lactis UBBLa‐70 decreased serum lipids in obese women on a low‐caloric diet, while this probiotic in combination with fructooligosaccharides (FOS) had a modulating effect on the metabolome but no effect on the lipid profile. Neither intervention had a measurable effect on weight, BMI or the microbiome.^78^ Bifidobacterium animalis subsp. lactis 420 in combination with a prebiotic modulated the microbiome of overweight adults, a circumstance, which was mainly driven by the prebiotic component while the probiotic without prebiotic showed only minimal effects on the microbiome composition and function.^79^ An intervention with either B. adolescentis IVS‐1 or B. animalis BB‐12 with or without galactooligosaccharides increased the abundance of the probiotic strains as well as the parent taxa in the microbiome to various degrees but had no effect on the gut permeability of overweight adults in a short term 3‐week intervention.^80^ In combination with a very low‐caloric ketogenic weight loss program, a single‐strain synbiotic containing B. animalis subsp. lactis was more effective in regards to weight loss and reduction of gamma gutamyl transferase than a multi‐strain synbiotic given in succession with the single‐strain synbiotic or a placebo. This intervention was associated with higher levels of Porphyromonadaceae, Christensenellaceae, Parabacteroides and Lachnospira.^81^

### 2.9 Multi‐strain/multi‐species probiotics

A probiotic containing *B. longum* BB536 and *L. rhamnosus* HN001 in combination with a Mediterranean diet reduced waist circumference, waist‐to‐hip ratio and insulin levels, while increasing alpha diversity, Eubacterium, L‐Ruminococcus and decreasing Bacteroides and Butyricicoccus in the microbiome compared with a Mediterranean diet alone in obese female breast cancer survivors.^82^ The combination of two *B. breve* strains (BR03 and B632) also reduced waist circumference, the BMI standard deviation score, fasting insulin and ALT in obese children and adolescents in a weight loss program, while the changes in the microbiome were similar to the placebo group.^83^ *L. curvatus* HY7601 in combination with *L. plantarum* KY1032 improved body weight and composition and increased adiponectin levels in otherwise healthy overweight and obese adults. The probiotic and the placebo group underwent changes in the microbiome, including an increase in *A. muciniphila* and a decrease of *F. prausnitzii* in the probiotic group.^84^

VSL#3, one of the most researched probiotic consortia, increased body weight and adiposity in Latino adolescents without exerting changes on the microbiome^85^ and increased liver fat content, indicating that the choice of probiotic strains is crucial.^86^ A similar consortium also containing *Streptococcus thermophilus*, *Lactobacillus* spp. and *Bifidobacteria* spp. (for details see Table 2) increased HDL levels and probiotic bacteria in the microbiome of overweight women taking Bofutsushosan, a Japanese Kampo against obesity, but did not further influence metabolic parameters or the overall microbiome composition compared with women treated with Bofutsushosan alone.^87^ A Streptococcus/Lactobacilli/Bifidobacteria consortium with *Saccharomyces boulardii* DBVPG 6763 showed no effect on metabolic parameters compared with placebo; microbiome changes were reported for both groups.^88^ Microbiome changes in both groups were also observed in obese women treated with a Lactobacilli/Bifidobacteria consortium or placebo.^89^ Another Lactobacilli/Bifidobacteria combination modestly decreased HbA1c, fasting insulin and HOMA‐IR, while increasing Lactobacillus and Bifidobacterium counts in the microbiome (no further analysis was performed).^90^ Postmenopausal women showed improvements in lipopolysaccharides (or lipopolysaccharide levels), waist circumference, fat mass, subcutaneous fat, uric acid, total cholesterol, triglycerides, low‐density lipoprotein cholesterol, glucose, insulin and HOMA‐IR after a 12‐week intervention with a Lactobacilli/Bifidobacteria/Lactococci formulation, although the modulation of the microbiome structure and function was minimal.^91^, ^92^ The same probiotic combined with FOS and GOS decreased hip circumference and lipoprotein (a) and improved quality of life, also with minimal impact on the microbiome structure of obese type 2 diabetic patients.^93^ A synbiotic consortium with considerable overlap in probiotic strains used in^91^, ^93^ combined with FOS, improved zonulin levels and decreased proteolytic bacteria in the microbiome while increasing total bacterial count in overweight adults.^94^ A Lactobacilli‐Bifidobacteria‐GOS combination in addition to a weight loss program reduced HbA1c levels in obese adults, while altering several taxa of the microbiome (for details see Table 2).^95^ Conversely, a combination of LcS and *B. breve* YIT 12272 (BbrY) with GOS increased HbA1c and fasting glucose in patients with type 2 diabetes while modulating the microbiome, especially taxa taxonomically related to the ingested probiotic strains.^96^ Bacteroides fragilis to *E. coli* ratio increased and Firmicutes to Bacteroidetes ratio decreased in prediabetic patients after a 24‐week intervention with a Lactobacilli/Bifidobacteria/inulin‐formulation.^97^

In the currently largest clinical trial testing a probiotic consortium in newly diagnosed type 2 diabetes patients, neither a metabolic nor a microbiome modulating effect of a standalone probiotic intervention was observed.^98^ In a secondary analysis, a synergistic effect of the multi‐species probiotic and berberin on postprandial cholesterols and other lipidomic parameters was found.^99^ Unfortunately, the trial included a study‐specific 7‐day run‐in period with antibiotics, which influenced the microbiome composition, which is of questionable clinical usability considering the antibiotic‐resistance crisis.

### 2.10 Prebiotics

Prebiotics are defined as ‘substrates that are selectively utilized by host microorganisms conferring a health benefit’.^100^ Since the recognition of their beneficial physiological effect over two decades ago, the interest in prebiotics has grown markedly. Currently, the best‐known prebiotics are fructo‐oligosaccharides, inulin, oligofructose, galacto‐oligosaccharides and resistant starch, wich for example demonstrated the promotion of growth of Bifidobacteria and Lactobacilli.^101^ Since the gut microbiome in people with excessive weight shows a reduction in diversity of bacteria in general and specifically a reduction of the abundance of Bifidobacteria, using prebiotics to restore gut microbiome ecology and function has been considered an attractive therapeutic option/method.^102^ Inulin, resistant starches or fructo‐oligosaccharide‐enriched inulin may have beneficial effects on components of MetS.^102^ Supplementation of trans‐galactooligosaccharides (GOS) improved insulin and lipid metabolism, increased the abundance of Bifidobacteria and decreased Gram‐negative bacteria such as *Bacteroides* spp.*, Desulfovibrio* spp. and the *C. histolyticum* group.^103^ Alpha‐galacto‐oligosaccharides led to a dose‐dependent decrease of appetite and food intake.^104^ Supplementation of GOS selectively increased the abundance of *Bifidobacterium* species, but did not lead to significant changes in peripheral insulin sensitivity, energy and substrate metabolism in prediabetic patients.^105^ Inulin‐type fructan supplementation decreased fat mass slightly, but did not significantly change BMI, lipid or glucose homeostasis.^106^ Inulin also favourably influenced microbiome composition.^107^, ^108^ While in adults no convincing effect on body weight could be observed, in obese children inulin and dietary fibre together with increased exercise showed a significant decrease in weight and body fat.^109^ Resistant starch type 4 (RS4) supplementation positively affected lipid metabolism, potentially by modulating bile acid metabolism and microbiome composition, but not glucose metabolism.^110^, ^111^, ^112^ The addition of polyphenolic food components increased butyrate‐producing gut bacteria and influenced the composition of the microbiome; however, the effects were largely dependent on the concomitant medication of patients.^113^, ^114^, ^115^ Taken together, prebiotics seem to modify gut microbiome composition but the effects on metabolic syndrome markers and body weight are rare. It is still unknown, which factors, such as timing, dose, treatment duration, type of the intervention and combination with other, well‐proven, interventions influence the effectiveness. The included clinical studies are summarized in Table 3.

**TABLE 3:** Clinical studies on prebiotics and the gut microbiome in patients with metabolic syndrome.

Study	Participants	Prebiotic intervention	Effect of dietary intervention on metabolic outcomes	Effect of dietary intervention on microbiome	Adverse events
Vulevic, et al., 2013^103^ Registered at clinicaltrials.gov NCT01004120	45 overweight adults with ≥3 risk factors associated with metabolic syndrome	12‐week supplementation (5.5 g B‐GOS/d), 4 weeks washout before another 12‐week supplemented diet versus placebo (maltodextrin)	Increased: sIgA, Decreased: plasma CRP, insulin, calprotectin. Decreased: plasma TG, TC and TC:HDL‐C ratio. No changes: G‐CSF, IL‐6, IL‐10, IL‐8, and TNFa, HDL‐C and LDL‐C	Increased: *Bifidobacterium* spp. Decreased: *Bacteroides* spp., *C. histolyticum* group, *Desulfovibrio* spp.	Not reported
Canfora et al., 2017^105^ Registered at clinicaltrials.gov NCT02271776	44 overweight or obese prediabetic adults aged 45–70 years, BMI = 28–40 kg/m^2^	12‐week supplementation (15 g/day of GOS) or placebo (maltodextrin)	No changes: peripheral insulin sensitivity, insulin, insulin‐stimulated FFA suppression, plasma and faecal SCFA, plasma glucose, glycerol, TAG, leptin, PYY, GLP‐1, IL6, IL8, TNF‐a, LBP, BMI, body weight, body fat percentage, body fat mass, lean mass, visceral adipose tissue mass, food intake, energy expenditure, fat and carbohydrate oxidation	Increased: *Bifidobacterium* spp. Small and inconsistent changes in *Prevotella oralis et rel*., *Prevotella melaninogenica et rel., Bacteroides stercoris et rel.,* and *Sutterella wadsworthia et rel*. No changes: Microbial richness and diversity	Not reported
Dewulf et al., 2013^106^ Registered at clinicaltrials.gov NCT00616057	30 obese women aged 18–65 years, BMI >30 kg/m^2^	3‐month supplementation (8 g/day during the first week and then 16 g/day of inulin/oligofructose 50/50 mix) or placebo (maltodextrin).	Decreased: post‐OGTT glycaemia No changes: BMI, waist/hip ratio, HbA1c, fasting glycaemia and insulinaemia, post‐OGTT insulinaemia, HOMA index, adiponectin, cholesterol, triglycerides, plasma CRP	Increased: Firmicutes, Actinobacteria, bacilli, Clostridium clusters IV and XVI, *Bifidobacterium, Faecalibacterium prausnitzii, Lactobacillus* spp. Decreased: Bacteroidetes, *Bacteroides intestinalis, Bacteroides vulgatus, Propionibacterium*	At the beginning of the supplementation six participants in the placebo group and 14 participants in the treated group experienced side effects (slight bloating, flatulence, and/or abdominal pain) a few days
Druart et al., 2014^116^ Registered at clinicaltrials.gov NCT00616057 Parent clinical trial – Dewulf et al., 2013^106^	30 obese women aged 18–65 years, BMI >30 kg/m^2^	3‐month supplementation (16 g/day of inulin/oligofructose 50/50 mix) or placebo (maltodextrin)	No changes: CLA, CLnA	No changes: *Roseburia* spp.	At the beginning of the supplementation 6 participants in the placebo group and 14 participants in the treated group experienced side effects (slight bloating, flatulence, and/or abdominal pain) a few days
Salazar et al., 2015^117^ Registered at clinicaltrials.gov NCT00616057 Parent clinical trial – Dewulf et al., 2013^106^	30 obese women aged 18–65 years, BMI >30 kg/m^2^	3‐month supplementation (16 g/day of inulin/oligofructose 50/50 mix) or placebo (maltodextrin)	Decreased: total SCFA, acetate, propionate	Increased: *B. adolescentis*, *B. longum*, *B. pseudocatenulatum*	At the beginning of the supplementation 6 participants in the placebo group and 14 participants in the treated group experienced side effects (slight bloating, flatulence and/or abdominal pain) a few days
Gonzales‐Sarias et al., 2018^115^ Registered at clinicaltrials.gov NCT02061098	49 overweight‐obese adults over 40 years, BMI >27 kg/m^2^	Supplementation in cross‐over fashion and dose–response: Dose 1 – one capsule/d‐450 mg PE (3 weeks), Dose 2–4 capsules/d‐1,8 g PE (3 weeks) and placebo (maltodextrin). Washout period: 3 weeks between the doses	Decreased: plasma LBP and hsCRP	Increased: Bacteroidetes, *Bacteroidaceae, Porphyromonadaceae, Bacteroides, Faecalibacterium, Parabacteroides, Odoribacter, Butyricimonas*, *Coprobacter* Decreased: Firmicutes, Euryarchaeota, Firmicutes/Bacteroidetes ratio, *Peptostreptococcaceae, Clostridiaceae, Coriobacteriaceae, Methanobacteriaceae, Romboutsia, Anaerostipes, Dorea, Clostridium* sensu stricto*, Butyricicoccus, Methanobrevibacter, Methanosphaera, Anaerofustis, Parvimonas*	Not reported
Cortes‐Martin et al., 2021^114^ Registered at clinicaltrials.gov NCT04075032	50 poly‐medicated metabolic syndrome patients (lipid‐lowering drugs, anti‐hypertensive drugs, oral anti‐diabetics) over 18 years, BMI >30 kg/m^2^	Two 4‐week supplementations in cross‐over fashion (900 mg/day of pomegranate extract nutraceutical) or placebo (microcrystalline cellulose) with a 4‐week washout period between the phases of the treatment	Decreased: sICAM‐1 (LL‐), LBP No changes: most of inflammatory and metabolic‐related markers, SCFA	Increased: *Bifidobacterium* (LL‐AD‐ groups, except HP‐); *Lactococcus* (LL‐, AD‐, HP‐ groups); *Hespellia* (AD‐, HP‐); *Weisella* (HP‐), *Buttiauxella* (AD‐); *Campylobacter* (LL‐), *Aestuariispira* (HP‐), *Alloscardovia* (HP‐), Tessaracoccus (non‐HP‐), Corynebacterium (non‐LL‐) Decreased: *Weisella* (LL‐), *Delfia* (AD‐), *Bacillus* (HP‐), *Clostridium XIVa* (non‐LL, non‐HP‐patients) No changes: *Weisella* (AD‐)	Not reported
Birkeland et al., 2020^108^ Registered at clinicaltrials.gov NCT02569684	25 patients aged 41–71 years with type 2 diabetes	Two 6‐week supplementations in cross‐over fashion (16 g/d of 50/50 mixture of oligofructose and inulin) and placebo (maltodextrin) with washout period of 4 weeks between supplementations	Increased: total SCFA, acetic and propionic acids No changes: butyric acid	Increased: at OTU level Actinobacteria, Bacteroidetes, Bacteroides, Clostridiales, *Bifidobacterium adolescentis*, *Bacteroides ovatus*, Lachnospiraceae, *Faecalibacterium prausnitzii*. Decreased: at OTU level Firmicutes, Ruminococcaceae, *Rumino‐ coccus,* Erysipelotrichaceae No changes: overall microbial diversity	16 participants in the treatment group and 2 participants in the control group reported passage of gas and flatulence worse or much worse than before the supplementation (the information from the follow up study PMID: 34297363)
Tian et al., 2022 107 No registration in a public repository could be found	60 patients aged 35–65 with ≥3 risk factors associated with metabolic syndrome	6‐month supplementation (inulin, inulin + TCM or inulin + metformin)	—	In inulin group more abundant: Bacteroidetes, Bacteroidaceae, Ruminococcaceae, Bacteroides. In inulin + TCM group more abundant: Proteobacteria, Enterobacteriaceae, Veillonellaceae, Romboutsia. In inulin + metformin more abundant: Streptococcaceae, Streptococcus, Holdemanella.	Not reported
Nichenametla et al., 2014^112^ Registered at clinicaltrials.gov NCT01887964	86 participants aged ≥18 years with and without metabolic syndrome	Two 12‐week study periods (resistant starch type 4 enriched flour or control wheat flour ad libitum) with a 2‐week washout period	Decreased: mean TC total cholesterol, non‐HDL, HDL cholesterol in the With‐MetS group. Decreased: waist circumference, body fat in No‐MetS group Increased: 1% in fat‐free mass in all participants No changes: blood pressure, fasting glucose, postprandial glucose, HbA1C	—	Not reported
Upadhyaya et al., 2016^111^ Registered at clinicaltrials.gov NCT01887964 Parent clinical trial – Nichenametla et al., 2014^112^	20 participants >18 years with metabolic syndrome	Two 12‐week study periods (resistant starch type 4 enriched flour or control wheat flour ad libitum) with a 2‐week washout period	Increased: faecal SCFAs (butyrate, propionate, valerate, isovalerate, hexanoate), adiponectin levels Decreased: IL6, percent body fat, non‐HDL, HDL, TC No changes: TNFα, blood pressure, triglyceride levels	Increased: at OTU level *Bacteroides, Parabacteroides, Oscillospira, Blautia, Ruminococcus, Eubacterium, Christensenella,* Clostridial cluster XIVa, *Bifidobacterium adolescentis, (compared with baseline)* Decreased: Firmicutes/Bacteroidetes ratio, *Enterococcus casseliflavus, Streptococcus cristatus* (compared with control)	Not reported
Dhakal et al., 2022^110^ Registered at clinicaltrials.gov NCT01887964 Parent clinical trial – Nichenametla et al., 2014^112^	14 participants aged 33–69 years with metabolic syndrome, BMI = 26.7–40.37 kg/m^2^	Two 12‐week study periods (resistant starch type 4 enriched flour or control wheat flour ad libitum) with a 2‐week washout period	Increased: microbiota‐derived bile acids (total, taurocholic, taurodeoxycholic, glycochenodeoxycholic, glycodeoxycholic, deoxycholic)	—	Not reported
Visuthranukul et al., 2022^109^ Registered at clinicaltrials.gov NCT03968003	155 obese children aged 7–15 years, BMI >2 standard deviations above median	6‐month intervention: inulin group — 13 g/d of isocaloric oligofructose enriched inulin (10 g according to clinicaltrials.gov), placebo group — 11 g/d of isocaloric maltodextrin), fibre advice group (age‐appropriate fibre intake)	Increased in all groups: geometric mean IL‐6 (significantly higher in obese children with acanthosis nigricans), dietary fibre intake Decreased in all groups: BMI z‐score, fat mass index, percent body fat, trunk fat mass index, caloric and fat intake, geometric mean for IL‐1β, TNF‐α No changes: FPG, lipid profiles, ALT (within groups), faecal calprotectin (among the groups)	—	Not reported
Xu et al., 2021^113^ Registered at www.chictr.org.cn ChiECRCT‐20,180,139	187 mildly hypercholesterolemic participants aged 18–65 years	45 day‐period consumption of 80 g of oats or rice (control group)	Increased: plasma acetic, propionic acid Decreased: TC, LDL‐C No changes: isobutyric, butyric, isovaleric, valeric, hexanoic acid	Increased: *Akkermansia muciniphila, Roseburia;* relative abundance of *Dialister, Butyrivibrio, Paraprevotella* Decreased: unclassified *f‐Sutterellaceae*	Not reported
Morel et al., 2015^104^ No registration in a public repository could be found	88 overweight adults aged 18–45 years, BMI = 25–28 kg/m^2^	2 studies: 1. Dose‐effect study—250 mL bottled oolong tea with added 3, 6, or 9 g α‐GOSs or control group (dried glycose syrup) twice per day 2. Formulation effect study—250 mL bottled oolong tea with 6 g of α‐GOSs with content of DP2, DP3 or DP4 or control (dried glycose syrup) twice per day. Intervention period—14 days	Increased: fulness, satiety Decreased: hunger, desire to eat, prospective consumption, food intake, LPS, CRP	Increased: faecal bifidobacteria	Increased: The flatulence score in the dose‐effect study; stool frequency in formulation‐effect study compared with the control group

Abbreviations: (AD‐), anti‐diabetic drugs; ALT, alanine aminotransferase; B‐GOS, Bi^2^muno (galacto‐oligosaccharide mixture); BMI, body mass index; CLA, conjugated linoleic acid; ClnA, conjugated linolenic acid; CRP, C‐reactive protein; DP, degree of polymerization; FFA, free fatty acid; FPG, plasma glucose; G‐CSF, granulocytes colony‐stimulating factor; GLP, glucagon‐like peptide; HbA1c, haemoglobin A1c; HDL‐C, high‐density lipoprotein cholesterol; HOMA, homeostasis model assessment; (HP‐), anti‐hypertensive drugs; IL, Interleukin; LBP, lipopolysaccharide‐binding protein; LDL‐C, low‐density lipoprotein cholesterol; (LL‐), lipid‐lowering drugs; MetS, metabolic syndrome; OGTT, oral glucose tolerance test; out, operational taxonomic unit; PYY, peptide YY; SCFA, short‐chain triacylglycerol; sICAM‐1, soluble intercellular adhesion molecule‐1; sIgA, secretory IgA; TAG, triacylglycerol; TC, total cholesterol; TCM, traditional Chinese medicine; TG, triglyceride; TNFa, tumour necrosis factor alpha.

### 2.11 Faecal microbiota transplantation for metabolic diseases

FMT, the transfer of faecal matter from one individual to another with the aim to improve/restore the composition of the gut microbiome and thereby treat a disease, has gained much attention in the scientific field and in the general public. As a method that in principle dates back to China in the fourth century, its modern application took off in 2013. Until now the only routine application is the treatment of recurrent *Clostridioides difficile* infections. Additionally, many non‐infectious diseases have been extensively studied.^118^ The first indication of efficacy for FMT in human metabolic diseases was published in 2012, where in a pilot study in the Netherlands insulin resistance was improved in nine male adults with metabolic syndrome who received FMT from a lean donor, whereas no changes were observed in nine controls who received an autologous FMT.^8^ A potential effect on body weight was first suspected by a ‘complication’ of FMT for *Clostridioides difficile*, where an initially normal‐weight patient received an FMT from an obese donor and gained weight after FMT.^119^ While consecutive studies did not report such effects,^120^ the opposite strategy—increasing body weight of cachectic cancer patients through FMT from obese donors—also failed.^121^ Nevertheless, BMI in the normal range has been included into the strict selection criteria for stool donors.^122^

After these initial proof of concept trials, a number of clinical trials have been conducted, where researchers aimed to improve metabolism and reduce weight by FMT in obesity, metabolic syndrome, non‐alcoholic fatty liver disease and type 2 diabetes. However, especially in adequately powered, randomized, placebo‐controlled trials, mixed results with regards to improvement in metabolic parameters were reported. While most studies demonstrated changes in microbiome composition, clear effects on clinically important endpoints, such as body weight or insulin resistance are missing. The included trials with their clinical endpoints are summarized in Table 4. It is notable, that the method of FMT may play a role in its effectiveness. Oral capsules, which would be the preferred route of administration for both safety and logistical reasons, so far did not show substantial improvements in metabolic parameters, except when administered together with low fermentable fibres.^123^, ^124^, ^125^, ^126^ From a mechanistic point of view it is still not clear what the ‘effective agent’ in FMT is. For the treatment of *Clostridioides difficile* infections, sterile filtrated faecal preparations were similarly effective as conventional faecal preparations containing living microorganisms. This indicates that bacterial components, metabolites, or bacteriophages may mediate the effects of FMT. The results of a pilot study suggests that FMT treatment, in absence of previous antibiotic treatment, significantly changes the bacteriophage community of the recipients. However, changing the bacteriophage community in this trial was not sufficient to present a clinical improvement in all the individuals.^127^ Another study reported an influence of FMT on plasma metabolites related to lipid metabolism and DNA methylation status; however, a clear‐cut pathophysiological explanation for a potential mechanism to influence glucose metabolism could not be identified.^128^

**TABLE 4:** Clinical studies on faecal microbiome transplantation in patients with metabolic syndrome.

Study	Population/number of participants	FMT mode/dose/duration	Primary endpoint	Result metabolic	Result microbiome	Adverse events
Yu 2020^126^ NCT02530385.	24 adults, obesity and insulin resistance	6 weeks, oral capsules, once per week versus placebo	Insulin sensitivity	No change in insulin sensitivity	Shift towards donor microbiome	No significant difference to placebo, no severe adverse events
Allegretti 2020^123^ NCT02741518	22 adults, obesity	8 weeks, 2 doses, oral capsules versus placebo	Safety	No reduction in BMI	Shift towards donor microbiome	No significant difference to placebo
Rinott 2021^131^, ^132^ NCT03020186	90 adults, obesity or dyslipidemia, randomized to healthy dietary guidelines, Mediterranean diet, and green‐Mediterranean diet weight‐loss groups	Autologous transplantation of microbiome collected under diet, 100 capsules in 8 months versus placebo	Weight regain	Autologous FMT attenuated weight regain in combination with green Mediterranean diet	Green Mediterranean diet caused change in microbiome composition	No treatment attributable adverse events
Mocanu 2021^125^ NCT03477916	70 adults, obesity and metabolic syndrome	6 weeks, oral capsules, 1 dose + either high‐fermentable or low‐fermentable fibres versus placebo	Insulin sensitivity	FMT + low fermentable fibres improved insulin sensitivity	FMT + low fermentable fibres increased diversity, shift towards donor microbiome	No treatment attributable adverse events
Wilson 2021^137^ ACTRN12615001351505	87 adolescents, obesity	6 weeks, oral capsules, 1 dose versus placebo	BMI	No effect on BMI, reduction of abdominal obesity, resolution of metabolic syndrome at baseline	Greater dissimilarity between baseline and post treatment in FMT group versus placebo, increase in diversity in female participants	No treatment attributable adverse events
Craven 2020^138^ NCT02496390	21 adults, non‐alcoholic fatty liver disease	6 weeks, allogenic or autologous FMT in distal duodenum via endoscopy, 1 dose	Insulin resistance	No effect on insulin resistance, reduction of increased intestinal permeability	No changes	Not reported
Ng 2022^134^ NCT03127696	61 adult, type 2 diabetes	24 weeks, allogenic FMT via nasogastric tube, 3 doses, lifestyle intervention	Donor microbiome engraftment	FMT + lifestyle intervention reduced total and low‐density lipoprotein cholesterol and liver stiffness (secondary endpoints)	FMT + lifestyle intervention significantly better engraftment (primary endpoint)	No differences between groups, several cardiovascular events
Xue 2022^139^ No registration in a public repository	75 adults, non‐alcoholic fatty liver disease	4 weeks, allogenic FMT, 3 doses, 1 via colonoscopy 3 via enema, versus oral probiotics	Clinical efficacy and safety (not further specified)	No increase in liver fat content compared with probiotic group	Differing responses in lean and obese patients	Not reported
Ding 2022^140^ ChiCTR‐ONC‐17011792	17 adults, type 2 diabetes	12 weeks, allogenic FMT, 2 doses, transendoscopic jejunal tube, unblinded	Insulin resistance	Improvement in HbA1c, glucose, uric acid, increase in C‐reactive protein	Difference between responders and non‐responders	No adverse events
Su 2022^141^ ChiCTR2100051257	16 adults, type 2 diabetes	12 weeks, allogenic FMT + formula diet versus formula diet alone, oral capsules, 3 doses	Health status	Both intervention improved BMI, glucose metabolism and blood pressure	Reduction of diversity in both groups, less in FMT group	No adverse events

Recent studies also explored how the selection of the donor could impact on the effect of FMT. A vegan diet is associated with reduced trimethylamine‐N‐oxide (TMAO) production and therefore lower cardiovascular risk; however, the FMT from vegan donors did not decrease TMAO production in patients with metabolic syndrome, which indicates that despite existing evidence of compositional changes that resemble the microbiome of the donor, the functional capacity is not easily transferred.^129^ FMT from patients after bariatric surgery to obese patients with MetS revealed microbiome‐driven modulation of brain dopamine and serotonin transporters.^130^ Furthermore, the use of autologous faecal transplants with faecal material obtained at the ‘weight nadir’ of a successful diet was able to delay weight regain after the diet.^131^, ^132^ These studies indicate that the concept of FMT most likely needs to be augmented by adequate preparatory measures like dietary changes or additional prebiotic ‘fertilizers’ of the transplanted microbiomes. Since studies also show considerable differences between different donors, it is essential to characterize donors and understand the interaction between the dysbiotic microbiome of the recipient and the microbiome of the donor.^133^ Special dietary measures, such as fibre supplementation may improve functional engraftment of FMT.^125^, ^134^ A recent systematic review did not identify consistent changes in clinically relevant endpoints for metabolic diseases (such as insulin sensitivity) achieved in the recipient after FMT.^135^ When transferring living microorganisms to a new host, adverse events have to be considered. It is surprising that some studies do not report safety data (see Table 4). A meta‐analysis on the safety of FMT across different disease entities showed no significant differences in the incidence of adverse events between FMT and the control group. Adverse events can be related to the transplanted microbiome or to the route of administration. It seems that administration via oral capsules or endoscopically via the lower gastrointestinal tract is less prone to adverse events. Translocation and infection with transplanted bacteria can occur, the risk seems to be higher in patients with an altered intestinal barrier.^136^ In the studies related to metabolic diseases no events of bacteremia or sepsis have been described so far.

However, from a practical point of view, it is unlikely that FMT, which requires highly skilled personnel and is resource‐intensive, will be applicable to treat the worldwide ‘obesity pandemic’. Further efforts are necessary to improve the timely and personalized diagnosis of the individual dysbiosis in obesity and to augment and retain the effect of a diet by influencing the microbiome in a personalized but also ‘affordable’ microbiome modulation strategy.

### 2.12 Influence of drugs for the treatment of metabolic diseases on the gut microbiome

Common drugs used in the treatment of metabolic diseases include metformin, statins and GLP‐1 agonists. These drugs also affect the gut microbiome composition and the gut microbiome composition in turn is thought to affect their therapeutic effects. Therefore, this review summarizes the current knowledge about the interactions of the drugs used for the treatment of metabolic diseases with the gut microbiome.

While metformin is a widely used therapy to treat type 2 diabetes mellitus, it is in a bidirectional relationship with the gut microbiome.^142^ In an attempt to disentangle diabetes and the metformin related changes in the gut microbiome, a seminal study including 784 human gut metagenomes showed that metformin therapy increases *Escherichia* and lowers *Intestinibacter* species abundance,^143^ as has also been shown by other studies.^144^, ^145^, ^146^, ^147^ The increase in *Escherichia* may explain some of the side‐effects of metformin including an increase in virulence factors and gas metabolism genes.^143^ Furthermore, the functional profile of the microbiome showed an increased potential for the production of butyrate and propionate, which has beneficial effects on glucose and energy.^143^ An increase in the production of short‐chain fatty acids was also described by other authors/studies.^145^, ^147^ Moreover, mucin‐degrading and butyrate‐producing *Akkermansia muciniphila* and *Bifidobacterium adolescentis* were described to be increased through metformin.^147^, ^148^ Another study by Sun et al, 2018 showed the decreased abundance of *Bacteroides fragilis*, which is a bile acid metabolizing bacterium, in the group of metformin treated diabetic patients, and a prominent increase in glycoursodeoxycholic acid levels.^149^ The link to bile acid metabolism was strengthened by the finding of increased *Blautia* species abundance associated with metformin treatment, with Blautia species being involved in bile acid metabolism,^150^, ^151^ and by the finding of significantly increased levels of total, primary, secondary and unconjugated plasma bile acids, which significantly correlated with lower HbA1c levels.^152^ Generally several studies reported an association between metformin mediated changes of gut microbiome composition and the function and effects of metformin on glycemic control.^149^, ^153^ An association between adverse effects of metformin and microbiome composition was also reported.^146^, ^154^ Therefore, metformin seems to influence microbiome composition and function, which might explain both adverse and beneficial effects of metformin treatment. Furthermore, tolerance and response to metformin were also associated with gut microbiome composition. Thus, patients with a higher abundance of *Megamonas rupellensis* and *Phascolarctobacterium* spp., a higher activity of the amino acid biosynthesis pathways and a lower activity of sugar degradation pathways before the start of a metformin therapy, were more tolerant to a subsequent metformin therapy.^155^ Maintenance of optimal glycemic control with metformin therapy in type 2 diabetes mellitus patients was associated with reduced alpha diversity and a peculiar signature of microbiome composition and functional pathways.^156^ Response to metformin was associated with a higher abundance of *Enterococcus faecium, Lactococcus lactis, Odoribacter* and *Dialister* before the start of metformin treatment in patients with type 2 diabetes mellitus.^154^

Statins are known to have broad effects not only in regards to metabolic syndrome but also beyond.^157^ Hu et al., 2021 reported that statin use in acute coronary syndrome shifts gut microbiome composition and function to a ‘healthier’ one, through, for example, a decrease in *Parabacteroides merdae* and an increase in *Bifidobacterium longum* subsp. *longum*, *Anaerostipes hadrus* and *Ruminococcus obeum* in the gut microbiome, which correlated with fatty acid and isoprenoid‐related pathways and promoted statin‐related beneficial metabolic effects.^158^ However, some studies showed conflicting results, for example, regarding the effect on Bacteroides abundance in patients treated with statins with some reports of increased Bacteroides^159^ and some reports of decreased Bacteroides,^160^ which might be attributed to, for example, different statin types predominantly used for treatment.^159^ Some studies show that there is no statin associated microbiome change,^161^ while others describe a whole range of altered bacterial taxa, also indicating the importance of standardization of microbiome sequencing and data analysis.^162^ Similar to metformin, statin treatment efficacy was linked to microbiome composition. Rosuvastatin was associated with differences in gut microbiome composition in patients with hyperlipidemia, with *Lactobacillaceae* and *Bifidobacteriaceae* being more abundant in patients with more pronounced effects of rosuvastatin.^163^, ^164^ Another study connected more successful statin treatment to a higher abundance of *Akkermansia muciniphila* and *Lactobacillus* and a lower abundance of *Holdemanella* and *Facecallibacterium*.^152^ A better response to atorvastatin was associated with a higher relative abundance of *Lactobacillus*, *Eubacterium*, *Faecalibacterium* and *Bifidobacterium*, but a lower relative abundance of *Clostridium*.^165^

Glucagon‐like peptide‐1 agonists are another group of drugs currently used in therapy of metabolic diseases. However, the data on its associations with the gut microbiome composition are so far relatively scarce. Interestingly, when comparing effects of metformin and GLP‐1 agonists on the gut microbiome, GLP‐1 agonists seem to have a stronger effect on increasing *Akkermansia* abundance than metformin.^166^ However, another study reported no effects of the GLP‐1 agonist liraglutide on the composition of the gut microbiome, despite its metabolic effects. The reason for contrasting results could be that Wang et al. 2018 did not account for the differences in pre‐treatment microbiome composition in patient groups in their analysis. The GLP‐1 agonist liraglutide was also shown to increase faecal abundance of deoxycholic bile acid.^167^ Diabetic patients who responded better to GLP‐1 agonist (liraglutide or dulaglutide) treatment were characterized by a specific microbiome signature, including an increased abundance of *Bacteroides dorei*, *Lachnoclostridium* sp., *Roseburia inulinivorans*, *Butyricicoccus* sp. and a decreased abundance of *P. copri*, *Bacteroidales*, *Ruminoccoccaceae*, *Eubacterium coprostanoligenes* sp., *Dialister succinatiphilus*, *Alistipes obesi*, *Mitsuokella* spp., *Butyricimonas virosa*, *Moryella* sp. and *Lactobacillus mucosae* compared with non‐responders.^168^ This suggests that the composition of the gut microbiome influences the efficacy of the treatment with GLP‐1 agonists. Contrasting and scarce findings on GLP‐1 agonist interaction with the gut microbiome urge further investigation of this topic. More details of the studies about the influence of the above described drugs on the gut microbiome are presented in Table 5.

**TABLE 5:** Human studies on the influence of metformin, statins and GLP‐1 agonists on the gut microbiome.

Study	Treatment	Participants	Alpha‐diversity	Increased bacterial taxa	Decreased bacterial taxa
Gradisteanu Pircalabioru 2022^169^	Metformin	40 subjects with metabolic syndrome	Decreased richness	Rikenellaceae RC9 gut group	Prevotella 9 Bacteroides Prevotellaceae Clostridiales
Deng 2022^170^	Metformin, 3 months 1700 mg/d	76 patients with treatment‐naïve type 2 diabetes, 36 patients are treated with metformin	Did not change	Ruminococcocaea Christensenellacaeae Blautia Roseburia Butyricimonas Lachnospira Clostridiales Oxalobacter Butirocycoccus Klebsiella Leuconostoc	Lachnospiraceae Romboutsia Clostridium Terrisporobacter Intestinibacter Streprococcus Ruminococcocaea Flavonitractor Erysipelatoclostridium
He 2022^171^	Metformin, 2 months, started at a dose of 500 mg/d and increased progressively during the first week to a dosage of 2000 mg/d	3 patients with treatment‐naïve type 2 diabetes	Did not change	None	Megamonas Klebsiella
Molina‐Vega 2022^172^	Metformin	58 women with gestational diabetes mellitus, 30 of them received metformin	Lower phylogenetic diversity	Enterobacteriaceae C. catus	Peptostreptococcaceae
Lee 2021^144^	Metformin, first dose 500 mg, 1000 mg twice daily for 4 days	20 healthy male subjects	Increased (Shannon)	Escherichia	Intestinibacter Clostridium Romboutsia
Mueller 2021^145^	Metformin, up to 2000 mg for 12 months	Overweight/obese adults treated for solid tumours, 42 patients on metformin treatment	—	*Escherichia coli* Ruminococcus torques	Intestinibacter bartlettii Roseburia faecis Roseburia intestinalis
Kim 2021^153^	Metformin, first dose 500 mg, 1000 mg twice daily for 4 days	10 healthy male subjects	Did not change	Escherichia	Parabacteroides
Tian 2021^173^	Metformin	Patients with type 2 diabetes mellitus and stable coronary artery disease, 18 patients treated with metformin	Increased gene richness	Unclassified Clostridium spp.	Prevotella bryantii Citrobacter koseri Acidaminococcus fermentans
Alvarez‐Silva 2021^174^	Metformin	Type 2 diabetes patients, 166 received metformin	Decreased richness	Bacteroides	Faecalibacterium
Elbere^154^	Metformin, dose 2 × 850 mg/day for 7 days or dose determined by an endocrinologist	35 healthy nondiabetic individuals and 50 newly diagnosed type 2 diabetes patients	Decreased in healthy controls, but not in patients	Parabacteroides distasonis Oscillibacter unclassified	Clostridium bartlettii Barnesiella intestinihominis
Zhang 2019^175^	Metformin, more than 3 months	Type 2 diabetes mellitus patients,	—	Spirochaete Turicibacter Fusobacterium	None
Bryrup 2019^146^	Metformin, 500 mg once daily for the first week, 500 mg twice daily for the second week, 1000 mg + 500 mg daily for the third week and 1000 mg + 1000 mg daily for the remaining 3 weeks of the intervention period	27 healthy young men	Did not change	Escherichia/Shigella sppBilophila wadsworthia Lachnoclostridium Caproiciproducens Prevotella Tyzzerella (Tyzzerella_3)	Intestinibacter bartletti Clostridium Terrisporobacter Senegalimassilia
Sun 2018^165^	Metformin, 1000 mg metformin twice daily (b.i.d) for 3 days	22 patients with type 2 diabetes mellitus	Slighly dicreased (Shannon)	None	Bacteroides (highest decrease in B. fragilis)
Tong 2018^150^	Metformin, 12 weeks of treatment, 0.25 g/time and 3 times/day orally after meals	Patients with type 2 diabetes mellitus and hyperlipidemia, 100 patients treated with metformin	Increased Simpson's diversity index	Blautia Clostridium XIVa Erysipelotrichaceae incertae sedis Escherichia/Shigella Fusobacterium Flavonifractor Lachnospiraceae Lachnospiracea incertaesedis Clostridium XVIII and IV Anaerostipes	Bacteroides Parabacteroides Alistipes Oscillibacter Ruminococcaceae
Huang 2018^176^	Metformin	Patients with type 2 diabetes mellitus, 23 patients were treated with metformin	—	Enterobacteriaceae	None
Wu 2017^152^	Metformin, 1700 mg/d for 4 months	Treatment‐naïve patients with type 2 diabetes mellitus, 22 patients were treated with metformin	—	Escherichia Bifidobacterium Akkermansia muciniphila Bifidobacterium adolescentis	Intestinibacter
de la Cuesta‐Zuluaga, 2017^148^	Metformin	Patients with type 2 diabetes mellitus, 14 patients were treated with metformin	Did not change	Prevotella Megasphaera A. muciniphila	Oscillospira Barnesiellaceae Clostridiaceae 02d06
Forslund 2015^143^	Metformin	Gut metagenomes of type 2 diabetes mellitus patients, 93 patients treated with metformin	Gene richness did not change	Escherichia spp.	Intestinibacter
Smits 2021^167^	GLP‐1 agonist (liraglutide (1.8 mg sc) once daily for 12 weeks	Patients with type 2 diabetes mellitus, 19 patients were treated with liraglutide	Did not change	Did not change	Did not change
Wang 2018^166^	GLP‐1 agonist (liraglutide) for 12 weeks and 18 weeks	Patients with type 2 diabetes mellitus, 19 patients were treated with liraglutide	—	Akkermansia unknown genus in the family Christensenellaceae	Sutterella
Wilmasnki 2022^159^	Statins	American adults cohort, European adults cohort with cardiometabolic diseases	Decreased (Shannon)	Bacteroides enterotype	—
Hu 2021^158^	Statins for more than 4 weeks	Patients with acute coronary syndrome, 36 patients were receiving statins	Did not change	Bifidobacterium longum subsp. Longum Anaerostipes hadrus Ruminococcus obeum	Parabacteroides merdae
Vieira‐Silva 2020^160^	Statins (48% simvastatin, 31% atorvastatin, 21% other statins)	MetaCardis Body Mass Index Spectrum cohort	—	—	Bacteroides enterotype
Kummen 2020^161^	Statins (rosuvastatin 20 mg daily for 6 months)	Women without obstructive coronary artery disease, 20 received rosuvastatin	Did not change	Did not change	Did not change
Khan 2018^162^	Statins (atorvastatin 20 mg dose for 2 years)	Hypercholesterolemic patients, 27 patients treated with atorvastatin	Decreased richness, decreased Shannon index	Firmicutes *Faecalibacterium prausnitzii* Akkermansia muciniphila genus Oscillospira *Ruminococcus* sp. *B. dorei B. uniformis* Bifidobacterium	Proteobacteria Desulfovibrio sp Bilophila wadsworthia Klebsiella, Streptococcus Collinsella *Bacteroides vulgates* *B. ovatus*

## 3 SUMMARY

Diet influences both metabolic diseases and the microbiome and changing dietary habits is a safe intervention. Clinical studies provide evidence that various dietary interventions, such as increases in whole‐grain or fermented foods, the Mediterranean diet or even the consumption of red wine improve some biomarkers of metabolic syndrome and change the composition of the gut microbiome. This gives hope to the hypothesis that the appropriate diet can influence the composition of the gut microbiome, and these changes can reduce the risk of the disease. However, the perfect diet has not been found yet—most likely, there will be no ‘one size fits all’ diet. The available literature on the use of probiotics in obese, MetS or diabetes patients is highly diverse in study designs, products, endpoints, microbiome analysis techniques and duration and dosage of interventions and therefore cannot support a sound conclusion of the efficacy of probiotics in obesity and related diseases. Prebiotics as a well‐defined form of dietary intervention, showed beneficial clinical effects for GOS, inulin and resistant starch on biomarkers of metabolic syndrome and in some cases these findings were associated with changes in the composition and function of the gut microbiome. FMT has so far not been able to show convincing clinical effects and the use of FMT as a strategy to treat obesity and metabolic diseases would also be limited by the fact that FMT is a resource‐intensive treatment that also contains some procedural risks. Changes in the composition of the gut microbiome that are associated with the intake of medications used to treat metabolic syndrome such as metformin, statins and GLP‐1 agonists and that can explain and modulate therapeutic and adverse effects of these drugs underpin the feasibility of managing the metabolic diseases with approaches targeting the microbiome.

## 4 LIMITATIONS

The heterogeneity of metabolic diseases and the complexity of the gut microbiome make it extremely difficult to deliver targeted microbiome modulation. A major shortcoming is for example the ubiquitous use of the umbrella term ‘probiotics’ which suggests a uniform intervention but includes vastly different bacterial species with potentially widely deviating functions. Although technically all discussed interventions are probiotics, there is no true replication and therefore validation of results. Similar patient cohorts have been treated with different products and vice versa, products used more than once have been trialled in different patient cohorts. This also limits the conclusion that can be drawn with regard to the involvement of microbiome modulation in the effects of probiotics. Some studies report clinical effects without microbiome modulation or microbiome modulation without resulting clinical effects. Furthermore, the techniques for microbiome analysis used in the discussed trials range from plating and counting over targeted qPCR to 16S sequencing, which massively influences the expectable resolution of results. Further research with evidence‐based formulations in adequately powered high‐quality randomized control trials is needed to improve the available data and support a sound conclusion on probiotics in obesity related diseases. Especially studies on prebiotics suffer from a small sample size as well as short and heterogeneous interventions, which limit the general applicability of these results in clinical practice.

## 5 FUTURE DIRECTIONS

To fulfil the promise of microbially derived therapies aimed at restoring metabolic health in humans, major efforts in translational science are needed to dissect the interaction of environmental influences on host‐microbe interplay. FMT clearly helped to understand the relationship between the gut microbiome and metabolic disorders and facilitated the notion that microbiome modulation can be an effective therapeutic strategy. However, unless developments such as encapsulated FMT are proven to be successful, the domain of FMT will be experimental rather than therapeutic. Furthermore, the fact that certain gut microbiome signatures in patients with metabolic syndrome are associated with the responce to these drugs highlights the urgent need for developing personalized gut microbiome targeting strategies not only to independently treat metabolic syndrome but also to ensure the efficacy of the currently used standard therapies via, for example, combination therapy. However, there are still a lot of unanswered questions regarding gut microbiome interactions with metabolic syndrome therapies, which need to be further investigated, including understanding the reasons for conflicting findings, the effects of combination therapies, and the influence of confounding variables on the results. The low predictability of the intervention warrants further research towards a better translation or transformation into clinical practice. Further efforts are necessary to improve a timely and personalized diagnosis of the individual dysbiosis in obesity and to augment and retain the effect of a diet by influencing the microbiome in a personalized but also ‘affordable’ microbiome modulation strategy. A clear commitment in research from all stakeholders (politics, funding bodies, health industry, researchers and the society) is necessary to move forward into the direction of developing live microbial agents, next‐generation probiotics and targeted dietary interventions to let the current hype develop into a realistic hope for patients with metabolic syndrome.

## AUTHOR CONTRIBUTIONS

**Angela Horvath:** Writing – original draft (equal); writing – review and editing (equal). **Kristina Zukauskaite:** Visualization (lead); Writing – original draft (equal); writing – review and editing (equal). **Olha Hazia:** Writing – original draft (equal); writing – review and editing (equal). **Irina Balazs:** Writing – original draft (equal); writing – review and editing (equal). **Vanessa Stadlbauer:** Conceptualization (lead), Writing – original draft (lead); writing – review and editing (lead).

## FUNDING INFORMATION

OH is supported by the ‘Crisis support for Researchers from Ukraine’, an Austrian Science Fund initiative, VS received funding from the Austrian Science Fund (KLI 741), and the project was partly conducted at the Center for Biomarker Research in Medicine (CBmed), a COMET K1 center funded by the Austrian Research Promotion Agency (Project 3.23).

## CONFLICT OF INTEREST STATEMENT

The authors have no conflicts of interest to declare.

## Data Availability

Not applicable.
